# Mode-Selective Tip-Enhanced Raman Spectroscopy Reveals Asymmetric Chemical and Carrier-Sensitive Raman Grading in Photovoltaic III–V Tandem Nanowire Junctions

**DOI:** 10.1021/acsnano.6c10214

**Published:** 2026-10-12

**Authors:** Vanessa Hinojosa-Chasiquiza, Jose Luis Pura, Siiri Bienz, Yue Zhao, Jordi Antoja-Lleonart, Magnus Borgström, Francesc Pérez-Murano, Sergio Muñoz de Baena, Renato Zenobi, Naresh Kumar, Jorge Serrano

**Affiliations:** † Gds-Optronlab, Dpto. Física de la Materia Condensada, 88193 Universidad de Valladolid, Paseo de Belén 19, 47011 Valladolid, Spain; ‡ Department of Chemistry and Applied Biosciences, 27219 ETH Zürich, 8093 Zürich, Switzerland; § Nano Lund and Division of Solid-State Physics, 5193 Lund University, Box 118, 221 00 Lund, Sweden; ∥ Hexagem AB, Ole Römers väg 1H, 223 63 Lund, Sweden; ⊥ Institute of Microelectronics of Barcelona (IMB−CNM, CSIC), Campus UAB, Bellaterra, 08193, Barcelona, Spain; # Wallenberg Initiative Materials Science for Sustainability, Department of Solid-State Physics, Lund University, 221 00 Lund, Sweden; ¶ Meeting Life LLC, 1210 Washington Ave. Ste. 213 Miami Beach, Florida 33139, United States

**Keywords:** InP/InGaP nanowires, axial heterojunctions, nanoscale interfacial analysis, tip-enhanced Raman spectroscopy, tandem solar cells

## Abstract

Performance of tandem junction nanowire (NW) solar cells relies on the electronic quality of the tunnel diodes which serve as the separating junctions between subcells. Yet, the relevant interfacial physics occurs below the optical diffraction limit. Here we use hyperspectral tip-enhanced Raman spectroscopy (TERS) to map, with nanoscale precision, axially defined heterojunctions in InP/InGaP tandem junction photovoltaic NWs. By resolving mode-specific phonon and phonon–plasmon responses, we show that the nominally abrupt p/p^++^ and p^++^/n^++^ junctions are compositionally graded. The TOM mode, used as the primary compositional marker, yields transition widths of ∼90–120 nm, whereas other lattice- and carrier-sensitive modes exhibit broader central widths up to ∼190 nm, reflecting their additional sensitivity to alloying, structural disorder, and electronic coupling. In a first-order approximation, rescaling these TERS-derived widths to shorter, device-relevant NWs yields narrower junctions, consistent with independent Kelvin probe force microscopy (KPFM) measurements that qualitatively capture the same trend despite surface and tip convolution effects. Complementary equilibrium band-edge model combined with a simplified one-dimensional Wentzel–Kramers–Brillouin (WKB) calculation, as an illustrative trend, further indicates that such grading is expected to widen the effective tunnel barrier. These findings establish mode-selective hyperspectral TERS as a validated, noninvasive method for quantitative nanoscale mapping of compositional grading in III–V NW junctions, with the carrier-sensitive L_1_ phonon–plasmon response providing complementary, model-assisted insight into the electronic evolution of the junctions.

## Graphical abstract

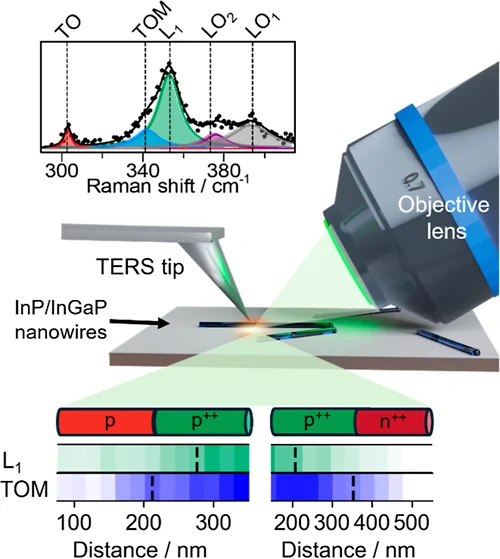

## Introduction

Semiconductor nanowires (NWs) provide a versatile platform for engineering complex axial and radial heterostructures with nanometer-scale precision.^1–3^ Their low-dimensional geometry facilitates strain relaxation and bandgap tunability, thereby enabling tailored electronic and optical properties that are attractive for advanced optoelectronic, photovoltaic, and power electronic applications.^4–7^ In photovoltaics, tandem solar cells comprising multiple subcells with distinct bandgaps are designed to harvest complementary portions of the solar spectrum, thereby surpassing the efficiency limits of single-junction devices. Optimal operation of tandem architectures requires subcells to be interconnected, typically by a tunnel diode, that is, a highly doped p–n junction that enables low-resistance carrier transfer via elastic tunneling, conserving both energy and momentum, in that way preventing current mismatch between subcells and ensuring high device performance.^8,9^ This principle applies equally to wafer-scale tandem devices and NW-based counterparts. For instance, Hrachowina *et al*. demonstrated double- and triple-junction III–V NW tandem solar cells incorporating tunnel diodes,^9^ where InGaP/InP NW devices achieved open-circuit voltages up to 1.91 V with power conversion efficiencies of ∼3.6%,^8^ while InGaP/InP/InAsP triple-junction devices exhibited an average open-circuit voltage of *V*_oc_ = 2.17 V.^10^

Efficiency losses in III–V NW photovoltaics largely arise from surface recombination, which reduces internal quantum efficiency, and from nonuniform or defective doping that generates carrier traps and limits charge separation. For instance, Hill *et al*. showed analytically that short-circuit current is strongly affected by surface recombination velocity,^11^ while studies on silicon NWs linked surface defects and doping-related traps to performance losses.^12,13^ Kim *et al*. further identified spatial doping inhomogeneity as a key limitation in III–V NW photovoltaics.^14^ Another key factor governing the performance of NW-based devices is the heterojunctions between segments of differing composition, such as tunnel diodes.^8^ These play a critical role in carrier transport,^15^ recombination dynamics,^16^ and band alignment.^17^ Tunnel junctions are rarely abrupt at the nanoscale since interdiffusion,^2,18,19^ growth kinetics,^20^ and dopant redistribution^21,22^ during synthesis often yield compositionally graded, spatially extended transitions that are challenging to probe using conventional analytical techniques. For example, atomic force microscopy (AFM) provides nanometer topographic resolution but lacks chemical sensitivity, while confocal Raman spectroscopy yields vibrational and structural information but is diffraction-limited to 200–300 nm.^23^ Since critical transitions in modern optoelectronic devices occur on sub-100 nm scales,^24^ neither technique can resolve the structural and electronic properties of NW heterojunctions.

Tip-enhanced Raman spectroscopy (TERS) overcomes the limitations of AFM and confocal Raman by combining nanometer-scale topographic resolution with chemically specific vibrational information.^25,26^ In TERS, localized surface plasmon resonance generated at the apex of a metallic AFM tip dramatically boosts Raman sensitivity and pushes the spatial resolution of Raman imaging to the nanoscale,^27,28^ making it ideally suited for probing semiconductor heterojunctions at the nanoscale. TERS has been successfully utilized for nanoscale chemical and structural characterization across diverse systems including defects in single-layer graphene,^24^ lattice deformations and strain in VO_2_ thin films,^29^ and interfacial diffusion in Sb_2_Se_3_-based solar cell thin films.^30^ Consequently, TERS offers a unique platform to investigate transition widths, vibrational mode evolution, and interfacial phenomena in semiconductor NWs for advancing tandem solar cell performance. However, its application to buried axial heterojunctions in III–V NW solar cells remains largely unexplored.

In this work, we employ nanoscale-resolved TERS to investigate InP/InGaP NW axial heterostructures engineered for tandem photovoltaic applications. These devices integrate an InP n-i-p bottom cell and an InGaP n-i-p top cell interconnected by a highly doped p^++^/n^++^ tunnel diode. Hyperspectral nanoscale-resolved TERS mapping reveals that the p/p^++^ (InP/InGaP) and p^++^/n^++^ (InGaP/InP) junctions are compositionally graded over 90–120 nm in an 8.4 μm long NW, with mode-dependent widths and distinct carrier-sensitive signatures. Independent Kelvin probe force microscopy (KPFM) measurements in a shorter device-relevant 2.7 μm long NW of the same growth batch, which probe the electronic potential at the surface, qualitatively confirm the trend of reduced transition widths. Such reduced transition lengths are qualitatively consistent with junction profiles that are more favorable for tunneling. A phonon–plasmon analysis based on an independently established Lindhard–Mermin calibration for the same tunnel-diode structure indicates that the free-hole concentration increases from the 10^17^ cm^–3^ range in the graded p-InP/p^++^-InGaP transition region to about 10^20^ cm^–3^ in the consolidated p^++^ segment, consistent with the nominal doping levels and with device-level electrical evidence^17,31^ of degenerate doping in equivalent NW tunnel diodes. Within the graded transition regions, where composition, disorder, and plasmon damping may vary spatially, the derived carrier-density profile is treated as a model-assisted consistency check with the nominal doping trend rather than as an independently validated electronic measurement. The earlier attenuation of the L_1_ phonon–plasmon Raman response, compared with the continued presence of composition-sensitive modes, is interpreted as a signature of changes in the local carrier/plasmonic environment near the junction, potentially arising from depletion effects, enhanced plasmon damping, alloy disorder, and/or variations in local optical coupling. A simplified Wentzel–Kramers–Brillouin (WKB) calculation, presented in the Supporting Information as an illustrative trend, further indicates why nanoscale junction grading is expected to reduce tunnel-barrier transparency. These findings provide guidelines for the rational design and nanoscale characterization of NW-based tandem solar cells and related optoelectronic devices.

## Results and Discussion

Figure 1a shows a schematic depiction of the TERS configuration used in this study. Representative spectra acquired from the same nominal location of an InP/InGaP NW are compared in Figure 1b under tip-engaged TERS and tip-out far-field conditions. For the far-field control measurement, the Ag-coated TERS probe was first retracted by approximately 10 nm from the TERS feedback position and the complete AFM tip holder was then physically removed from the optical path, while the sample position and optical focus were kept unchanged. Thus, no metallic tip was present near the NW during acquisition of the far-field spectrum, and no tip–sample near-field interaction could occur. Both spectra were acquired using the same low-power TERS-compatible conditions, namely an excitation laser power of 600 μW and an integration time of 10 s. Under these conditions, the far-field Raman signal from the NW is below the detection limit and no discernible vibrational bands are observed. In contrast, the tip-engaged TERS spectrum clearly resolves the characteristic Raman modes, demonstrating the strong plasmonic enhancement provided by the Ag-coated probe and confirming that the spectra used for nanoscale mapping are dominated by the tip-enhanced near-field contribution.

**Figure 1:**
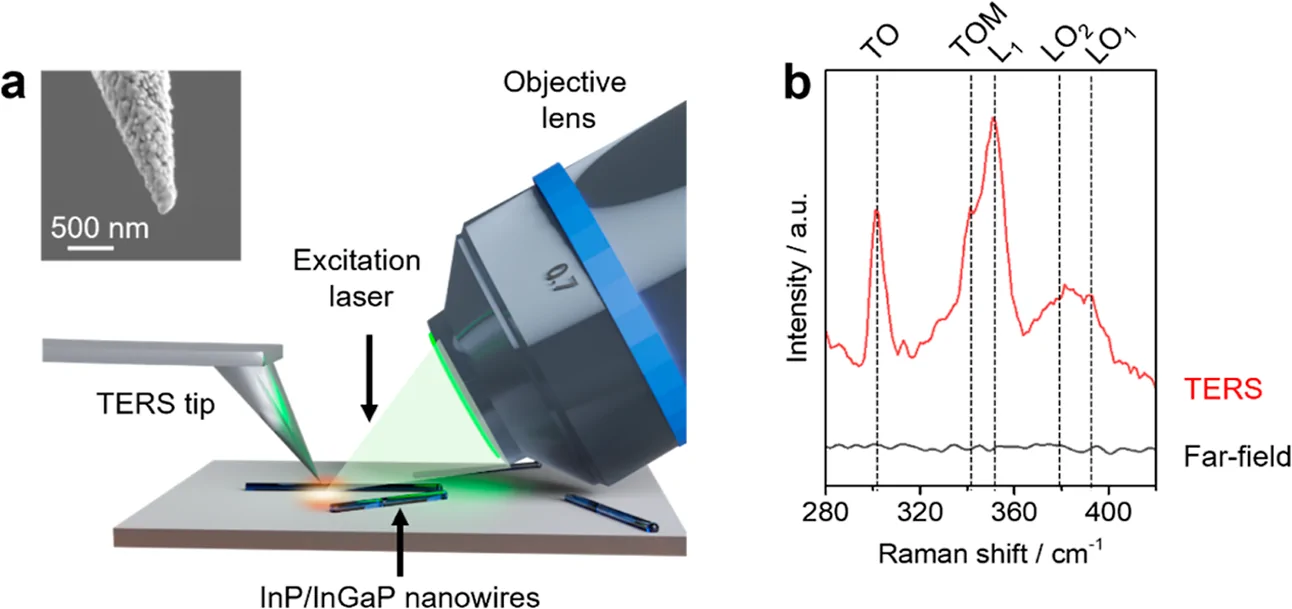
(**a**) Schematic illustration of the TERS setup used in this study. The inset shows a scanning electron microscopy (SEM) image of the Ag-coated AFM tip used for the measurements. A focused laser beam excites localized surface plasmons at the tip apex, generating a confined near-field that amplifies Raman scattering at the nanoscale. (**b**) Raman spectra acquired from the same nominal NW location under tip-out far-field control conditions, black, and tip-engaged TERS conditions, red. For the far-field spectrum, the Ag-coated tip was retracted and the AFM tip holder was physically removed from the optical path. Both spectra were measured with excitation laser power of 600 μW at the sample and spectrum integration time of 10 s.

Figure 2a shows a schematic illustration of the tandem axial architecture which comprises three regions: (i) an InP n-i-p bottom cell; (ii) a highly doped tunnel diode formed by p^++^-In_0.27_Ga_0.73_P (hereafter p^++^) and n^++^-InP (hereafter n^++^); and (iii) an In_0.91_Ga_0.09_P n-i-p top cell. For brevity, we denote the p-InP/p^++^-In_0.27_Ga_0.73_P junction as p/p^++^, and the p^++^-In_0.27_Ga_0.73_P/n^++^-InP tunnel junction as p^++^/n^++^. The AFM topography of the NW selected for TERS measurements is displayed in Figure 2b, where the InP segment appears on the left and the In_0.91_Ga_0.09_P segment on the right, identified by the terminal Au droplet. The length, height, and lateral size of the NW are determined to be ∼8.4 μm, ∼155 nm, and ∼540 nm, as measured by AFM, respectively (Figure S1). The value of ∼155 nm corresponds to the vertical height of the NW, whereas the apparent lateral width of ∼540 nm represents the convolution of the AFM tip apex and the rounded NW geometry. Moreover, the TERS tip, which is tilted with respect to the vertical axis by design, further contributes to this broadening effect. This is also reflected in the asymmetry of the topographic profile, as shown in Figure S1, panel (c).

**Figure 2:**
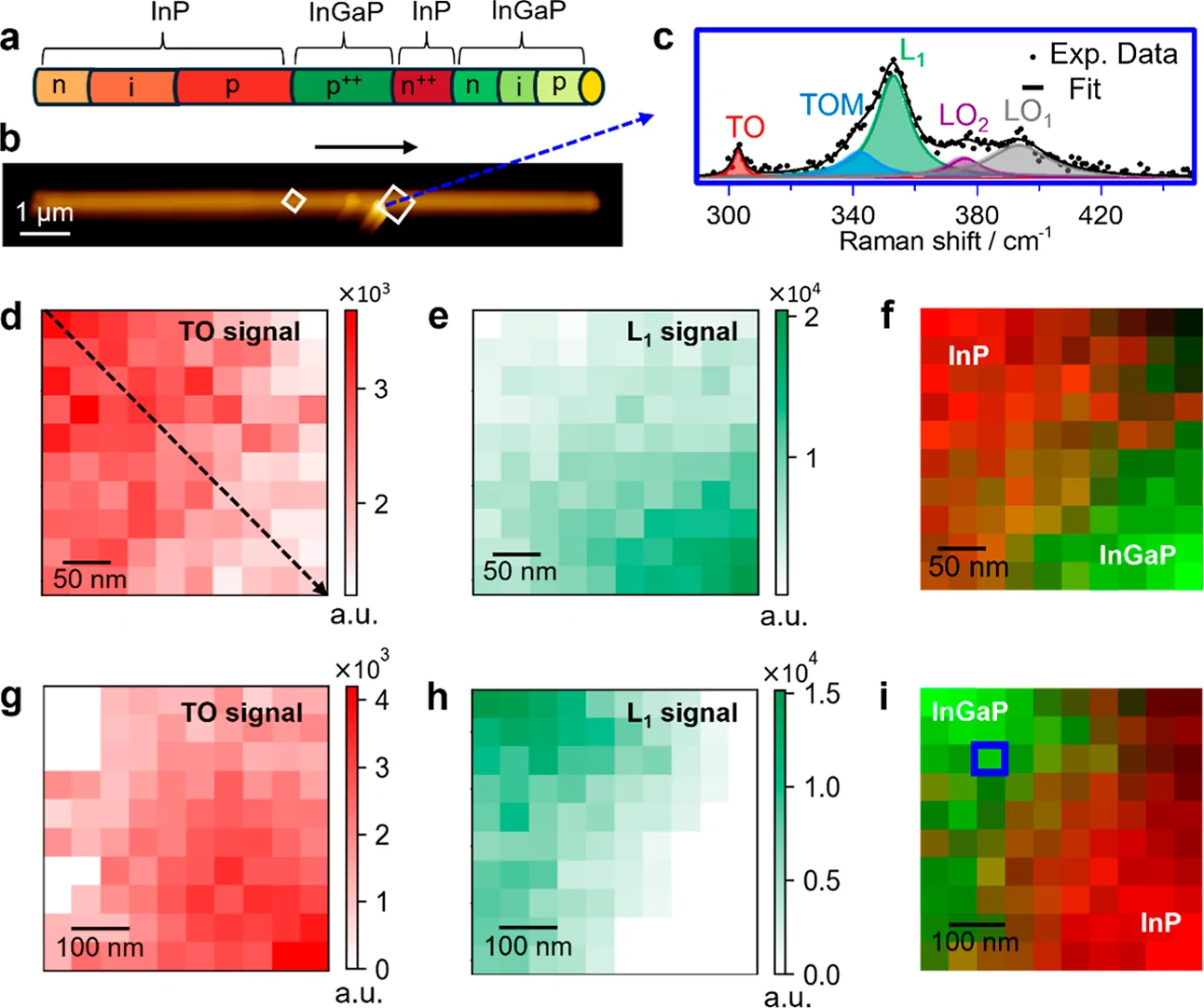
(**a**) Schematic of the InP/InGaP NW with labeled segments. (**b**) AFM topography image of the measured NW with a total length of 8.4 μm. The two marked squares indicate regions selected for TERS mapping. (**c**) Representative TERS spectrum acquired at the center of the tunnel diode. The location from which this spectrum was obtained is indicated by a blue dashed arrow in (**b**), and the corresponding pixel is highlighted by a blue box in (**i**). Shaded regions correspond to fitted components: red, TO mode (InP); blue, TOM mode (InGaP); green, L_1_ phonon–plasmon mode (InGaP), LO_2_ and LO_1_ modes of InGaP (purple and gray, respectively). Black dots represent experimental data, and the solid black line is the cumulative fit. (**d**–**f**) TERS maps at the p/p^++^ junction, the smaller square in panel b: (**d**) integrated intensity of the InP TO mode, (**e**) integrated intensity of the InGaP L_1_ mode, and (**f**) overlay of both maps showing spatial correlation. Step size: 30 nm. Spectrum integration time: 10 s. (**g**–**i**) TERS maps at the p^++^/n^++^ tunnel-diode junction (the bigger square at panel **b**) showing the same features as in (**d**–**f**): (**g**) InP TO, (**h**) InGaP L_1_, and (**i**) their overlay. Step size: 50 nm. Spectrum integration time: 10 s.

Although operational tandem junction cells employ 2–3 μm NWs, the longer NW investigated here, arising from near-edge overgrowth,^32^ provides finer axial sampling in hyperspectral TERS maps enabling more accurate interface profiling. Figure 2c shows a representative TERS spectrum extracted from the p^++^/n^++^ junction map (highlighted in blue in Figure 2i), where the characteristic phonon modes of InP and In_*x*_Ga_1–*x*_P are clearly resolved. The 303 cm^–1^ band is assigned to the first-order transverse optical (TO) phonon mode of InP, whereas the 342, 351, 374, and 389 cm^–1^ bands are assigned to the TO phonon (TOM), strongly damped longitudinal-optical phonon–hole-plasmon coupled mode (L_1_), and two longitudinal optical modes (LO_2_ and LO_1_) of In_*x*_Ga_1–*x*_P, respectively.^33^ These vibrational modes are consistent with confocal Raman measurements of the InP/InGaP NW acquired at higher laser power of 2.5 mW under top illumination conditions (Figure S2), confirming the preservation of the intrinsic vibrational response of the material in TERS measurements. The phonon frequencies of these modes vary with indium concentration and are described by the following equations^34–36^

1 $$\omega LO_{1}=404.99-38.97x-18.18x^{2}\left({cm}^{-1}\right)$$

2 $$\omega LO_{2}=394.59-80.36x+30.26x^{2}\left({cm}^{-1}\right)$$

3 $$\omega TOM=368.82-88.95x+26.04x^{2}\left({cm}^{-1}\right)$$

where *x = 1* for InP and *x = 0* for GaP, the parent compounds.

Table 1 compares the theoretical phonon frequencies with the experimental TERS results, showing good agreement and confirming the reliability of TERS for probing composition-dependent vibrational properties across different NW segments. The TO mode of InP is observed at slightly lower frequencies (301 cm^–1^ in the p-InP segment and 303 cm^–1^ in the n^++^-InP segment) compared to the nominal undoped value of ∼305 cm^–1^. This redshift arises primarily from lattice expansion and phonon softening induced by heavy doping and strain effects.^37^ In contrast, S doping on the P site in the n^++^-InP segment produces a smaller lattice expansion and therefore a less pronounced shift. Both effects are consistent with previous Raman studies on doped III–V semiconductors that showed a systematic reduction of TO phonon frequencies with increasing dopant concentration and associated lattice relaxation.^37–39^ The observed Raman shift difference between p- and n^++^-InP thus reflects the asymmetric lattice response to acceptor- and donor-type doping.

**Table 1:** Comparison of Theoretical Raman Mode Frequencies Calculated using eqs 1–3 and Experimentally Observed Raman Modes for Different NW Segments: p^++^-In_0.27_Ga_0.73_P, p-InP, and n^++^-InP

NW region	material	vibrational mode	calculated Raman shift (cm^–1^)	experimental Raman shift (cm^–1^)
p^++^	In_0.27_Ga_0.73_P	TO	347	--
		TOM	346	342
		L_1_	355	351
		LO_2_	375	374
		LO_1_	393	389
p and n^++^	InP	TO	306	301 (p), 303 (n^++^)
		LO_2_	345	--
		LO_1_	348	--

To probe the p/p^++^ and the p^++^/n^++^ tunnel diode heterojunctions along the NW, the integrated intensities of the InP TO mode and the In_*x*_Ga_1–*x*_P L_1_ mode were extracted by Lorentzian fitting. Figure 2d–f present spatial maps of the TO and L_1_ modes and their overlay across the p/p^++^ junction, while Figure 2g–i show the corresponding maps for the p^++^/n^++^ junction. The black arrow in Figure 2d marks the direction of the line profiles plotted in Figures 3 and 4, corresponding to the NW axial orientation. The spatial evolution of the Raman modes enables nanoscale visualization of compositional grading and junction transitions along the NW. For instance, at the p/p^++^ junction, the TO mode intensity (red) decreases as the p^++^ segment is approached (Figure 2d), while the L_1_ mode intensity (green) simultaneously increases (Figure 2e). In contrast, at the p^++^/n^++^ tunnel diode junction, the opposite trend is observed: the L_1_ signal decreases across the junction (Figure 2h), whereas the TO mode increases in intensity (Figure 2g). Notably, these variations are not abrupt; instead, the two vibrational modes overlap within a finite spatial region, as evident from the overlay of the TERS maps presented in Figure 2f and i. This coexistence indicates a gradual, rather than atomically abrupt, junction transition, consistent with compositional grading arising from adatom carry-over into the Au alloy catalyst (due to lower chemical potential) and the presence of residual group III species following the precursor switch.^40^ For clarity, only the TO and L_1_ modes are displayed in Figure 2, even though the In_*x*_Ga_1–*x*_P TOM mode exhibits the same spatial behavior as the L_1_ mode as shown in Figure S3.

**Figure 3:**
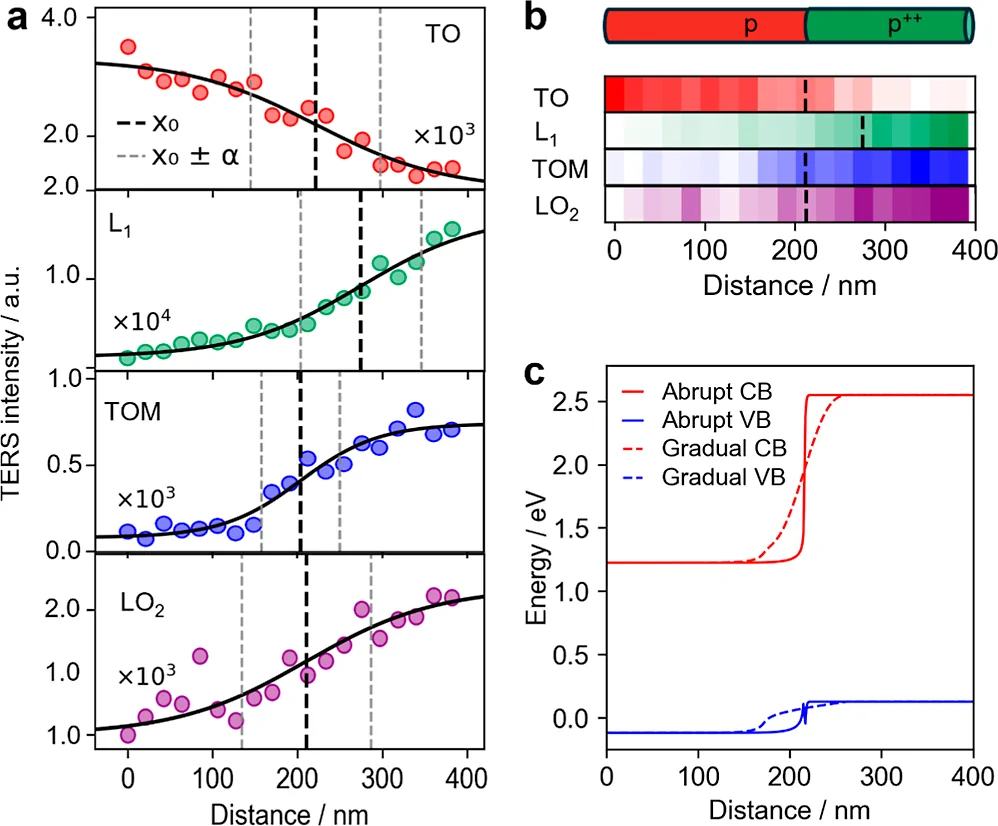
TERS line profiles across the p/p^++^ junction. (**a**) Integrated intensity profiles of the InP TO (red), In_0.27_Ga_0.73_P L_1_ (green), In_0.27_Ga_0.73_P TOM (blue), and In_0.27_Ga_0.73_P LO_2_ (purple) modes along the NW growth axis indicated in Figure 2d. Experimental data are shown as points, with sigmoid fits (eq 4) overlaid as black solid lines. The transition center (*x*_0_) and transition width (2α) for each mode are indicated by dashed black and gray vertical lines, respectively. (**b**) Schematic of the p/p^++^ junction of the NW with color-coded profiles of the TO (red), L_1_ (green), TOM (blue), and LO_2_ (purple) Raman modes. (**c**) Simulated band-edge profile across the p/p^++^ junction obtained with COMSOL Multiphysics. Red curves denote the conduction band (CB) and blue curves the valence band (VB). Solid lines represent an ideal abrupt junction, whereas dashed lines correspond to a graded junction using the experimental transition width (2α) extracted from the TOM fit in panel (**a**).

**Figure 4:**
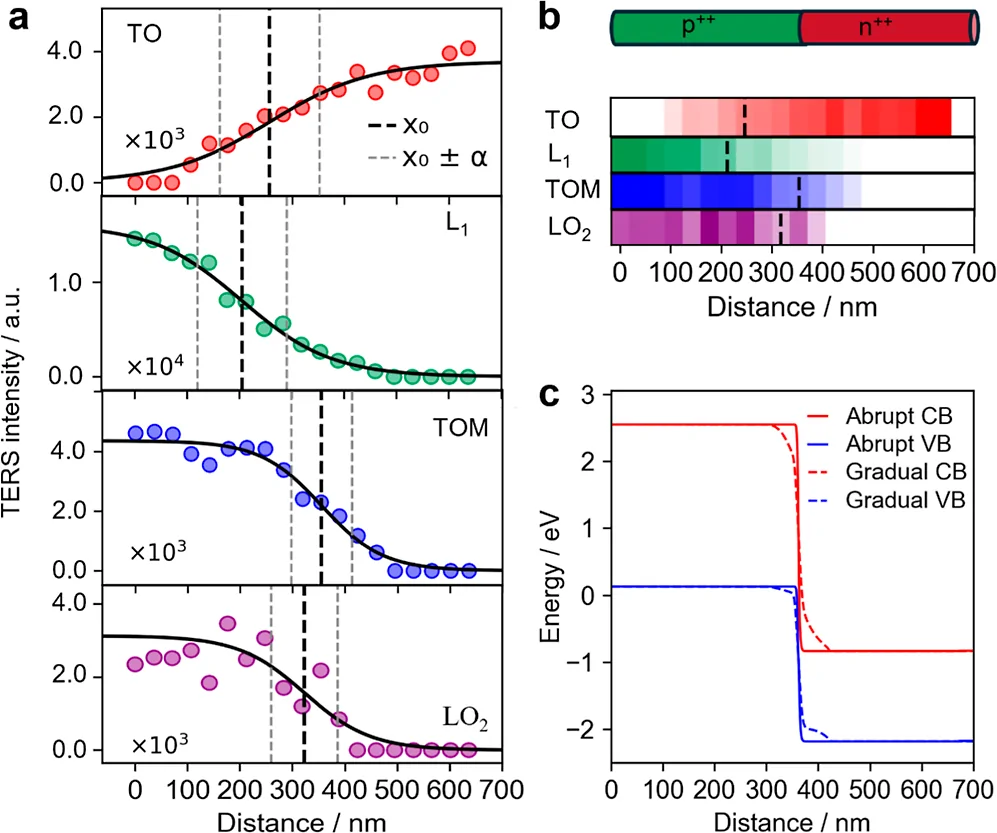
TERS line profiles across the p^++^/n^++^ tunnel diode junction. (**a**) Integrated intensity profiles of the InP TO (red), In_0.27_Ga_0.73_P L_1_ (green), In_0.27_Ga_0.73_P TOM (blue), and In_0.27_Ga_0.73_P LO_2_ (purple) modes along the NW growth axis. Experimental data are shown as points, with sigmoid fits (eq 4) overlaid as black solid lines. The transition center (*x*_0_) and transition width (2α) for each mode are indicated by dashed black and gray vertical lines, respectively. (**b**) Schematic of the NW structure highlighting the scanned p^++^/n^++^ junction, with color-coded profiles of the TO (red), L_1_ (green), TOM (blue), and LO_2_ (purple) Raman modes. (**c**) Simulated band-edge profile across the p^++^/n^++^ junction obtained with COMSOL Multiphysics. Red curves denote the conduction band (CB) and blue curves the valence band (VB). Solid lines correspond to an ideal abrupt junction, whereas dashed lines represent a graded junction using the experimental width (2α) extracted from panel (**a**).

To elucidate the structural characteristics of the p/p^++^ and p^++^/n^++^ junctions, line profiles of the integrated intensities of the TO, TOM, L_1_, and LO_2_ modes were extracted along the diagonal direction of the maps (marked in Figure 2d), corresponding to the NW growth axis. The complete stacked TERS spectra acquired along these lines, illustrating the continuous evolution of the Raman modes across each junction, are presented in Figure S4. To determine the transition center (*x*_0_) and transition width (2α) for each mode, these profiles were fitted using a sigmoid function (eq 4)­

4 $$I\left(x\right)=I_{\min}+\frac{I_{\max}-I_{\min}}{1+e^{\pm \left(x-x_{0}\right)/\alpha }}$$

where *α* describes the abruptness of the junction, and *I*_min_ and *I*_max_ represent the baseline and plateau intensities, respectively. The effective transition width, 2α, quantifies the spatial extent of the compositional or electronic transition depending on the mode analyzed. Note that L_1_ is a longitudinal-optical-phonon-hole-plasmon coupled mode and therefore reflects electronic characteristics of the system. In contrast, TO, TOM, and LO_2_ modes arise solely from the lattice vibrations and thus provide information primarily related to the local chemical environment.

The fitted *x*_0_ and 2α values are empirical descriptors of the measured spatial evolution of each Raman mode. For the lattice modes TO, TOM, and LO_2_, this evolution is interpreted primarily in terms of structural and compositional grading. The frequency and intensity of L_1_ mode are directly measured, but their conversion into free-hole concentration requires a phonon–plasmon coupling model and a damping parameter Γ previously determined in NWs containing tunnel diodes of the same composition as those studied here.^41^ In addition, the L_1_ intensity can be modified not only by changes in free-carrier density, but also by plasmon damping, alloy disorder, and local tip–sample optical coupling. We therefore use L_1_ as an electronic-sensitive marker of the local carrier/plasmonic environment, while treating carrier densities and electronic length scales derived from L_1_ as model-assisted, semiquantitative estimates.

Figure 3a shows the experimental intensity profiles and corresponding sigmoidal fits of the TO, L_1_, TOM, and LO_2_ Raman modes across the p/p^++^ junction, highlighting their nanoscale evolution. A schematic diagram of the p/p^++^ junction of the NW with color-coded profiles of the TO (red), L_1_ (green), TOM (blue), and LO_2_ (purple) modes is shown in Figure 3b. The Raman signals evolve gradually rather than abruptly, consistent with compositional and doping gradients governed by the growth dynamics at the tunnel diode junction. Interestingly, all composition-sensitive modes exhibit almost the same transition center, *x*_0_. The sigmoidal fits, with the combined fitting and spatial-sampling uncertainties described in Supplementary Note 1, yield transition centers of *x*_0_ = 220 ± 30 nm for the TO mode, *x*_0_ = 204 ± 18 nm for the TOM mode, and *x*_0_ = 210 ± 40 nm for the LO_2_ mode (Table 2). The values are given rounded to significant figures according to the calculated error. Overall, the chemically sensitive modes agree within uncertainty in the position of the transition. The corresponding effective transition widths are 2α = 150 ± 80 nm for TO, 2α = 90 ± 40 nm for TOM, and 2α = 150 ± 100 nm for LO_2_. The L_1_ mode gives *x*_0_ = 280 ± 30 nm and 2α = 140 ± 50 nm. Notably, the central transition widths are substantially larger than the independently validated ∼10 nm TERS near-field response, while the reported uncertainties account for the 30 nm spatial sampling of the TERS map. Thus, the profiles support a finite graded junction, although mode-to-mode differences should be interpreted within the combined uncertainties.

**Table 2:** Transition Centers *x*_0_ and Effective Transition Widths 2α Extracted from Sigmoidal Fits to the TERS Intensity Profiles Across the p/p^++^ and p^++^/n^++^ Junctions. The Reported Uncertainties Represent Combined Uncertainties Obtained by Quadratically Adding the Formal Fitting Uncertainty and the Spatial-sampling Uncertainty (Δ*x*). For *x*_0_, the Sampling Contribution was Taken as Δx/2, Whereas for 2α, a Conservative Contribution of Δ*x* was Used, where Δ*x* = 30 nm for the p/p^++^ Profile and Δ*x* = 50 nm for the p^++^/n^++^ Profile

p/p^++^			p^++^/n^++^	
	*x*_0_ (nm)	2α (nm)	*x*_0_ (nm)	2α (nm)
**TO**	220 ± 30	150 ± 80	260 ± 40	190 ± 80
**L**_**1**_	280 ± 30	140 ± 50	210 ± 30	170 ± 50
**TOM**	204 ± 18	90 ± 40	360 ± 30	120 ± 50
**LO**_**2**_	210 ± 40	150 ± 100	320 ± 40	130 ± 80

The L_1_ transition center is displaced relative to the TOM compositional marker by Δ*x*_*0*_ *=* 80 ± 40 nm, where the uncertainty is obtained by quadratically propagating the combined *x*_0_ uncertainties from Table 2. This displacement indicates that the spatial evolution of the L_1_ phonon–plasmon response does not coincide exactly with the structural transition tracked by TOM. We therefore interpret this offset as an electronic-sensitive signature of changes in the local carrier/plasmonic environment, rather than as a sharply defined electronic boundary or a direct measure of depletion width, since the L_1_ response may also be modified by plasmon damping, alloy disorder, and local optical-coupling variations near the graded interface.

This reflects a strong coupling between the InGaP LO_1_ phonon and the hole plasmon which is required to produce the -phonon–plasmon Raman signature. Such coupling only occurs for a consolidated InGaP composition, which corresponds to roughly *x*_0_ + α for the LO_2_ (InGaP) mode and overlaps with the L_1_ value for *x*_0_. These results highlight the capability of TERS to distinguish pure phononic signals from Raman signatures that arise from specific coupling effects with charge carrier-related quasiparticles. Figure S5 displays the predicted change in the L_1_ mode Raman shift as a function of hole concentration for different damping constants (Γ = 700, 900, and 1100 cm^–1^), adapted from the Lindhard–Mermin calculations reported by Mediavilla *et al*.^41^

The broader transition extracted from the TO mode can be explained by considering the lattice perturbation occurring during the growth of the p^++^-In_0.27_Ga_0.73_P segment. The incorporation of Ga and Zn into the growing NW lattice induces local strain and point defects due to differences in atomic radii and high dopant concentrations. These lattice perturbations preferentially affect the InP TO phonon, leading to peak broadening and reduced intensity. Consequently, the TO Raman mode yields a smoother, less abrupt integrated-intensity profile, which manifests as an apparently larger 2α transition width. Among the composition-sensitive phonons, the TOM mode provides the most reliable determination of the junction position. Although it appears as a shoulder in the Raman spectrum, its intensity seems to be less affected by strain- and defect-induced broadening, resulting in a sharper and more localized transition. This is consistent with the smaller uncertainties obtained for both *x*_0_ and 2α for the TOM mode.

In contrast, the LO_2_ mode exhibits significantly weaker signal intensity, resulting in larger fitting uncertainties, as reflected in its broader error ranges for both *x*_0_ and 2α. The LO_1_ mode, which could in principle serve as an indicator of the compositional transition, is even weaker and challenging to fit accurately; its intensity was not included in this transition center and transition width analysis. However, its peak position, determined with higher accuracy, is used in later sections to extract doping-related information. These observations reinforce the conclusion that the longitudinal optical modes of InGaP are generally poor markers of the p/p^++^ junction. In contrast, the TOM mode of InGaP tracks the actual Ga incorporation more faithfully, resulting in a smoother and spatially well-defined evolution along the NW axis. This behavior is consistent with previous Raman studies in III–V heterostructures, where alloying and heavy doping were shown to selectively perturb certain phonon modes. Accordingly, the TO mode serves as a sensitive indicator of lattice distortion and defect formation, while the TOM mode provides a more accurate measure of compositional grading across the junction.^42,43^ Thus, while the TO and LO_2_ modes clearly capture the chemical transition, the TOM mode offers the most precise and robust metric for locating the p/p^++^ compositional boundary.

The full set of fitted parameters for all modes at the p/p^++^ junction are summarized in Table 2. To evaluate the electronic consequences of this graded junction, band-edge simulations were performed with COMSOL Multiphysics using the experimental transition width. As shown in Figure 3c, the gradual compositional variation produces sloped band edges (dashed lines) in contrast to the abrupt step expected for an ideal junction (solid lines). This suggests that compositional grading can significantly impact carrier transport by affecting the tunneling recombination mechanisms within the transition width.

Unlike the p/p^++^ junction, the Raman response across the p^++^/n^++^ interface displays a markedly different behavior. The TOM and LO_2_ transition centers are *x*_0_ = 360 ± 30 nm and *x*_0_ = 320 ± 40 nm, respectively, and therefore agree within the combined uncertainty, indicating that both modes track the compositional transition across the p^++^/n^++^ junction. The TO mode displays an earlier transition center, *x*_0_ = 260 ± 40 nm, suggesting the onset of local InP-rich character before the full disappearance of the InGaP-related modes. The L_1_ mode exhibits an earlier transition, *x*_0_ = 210 ± 30 nm, consistent with its phonon–plasmon character and its sensitivity to the local free-carrier environment. Using the TOM mode as the compositional reference, the TOM–L_1_ transition-center offset is Δ*x*_0_ = 150 ± 40 nm. This offset remains resolved within the combined fitting and spatial-sampling uncertainty and is therefore interpreted as an electronic-sensitive signature of the junction region, consistent with previous measurements on comparable NW systems.^44,45^ However, we do not regard it as a direct quantitative measure of the depletion width. Rather, it reflects the spatial range over which the L_1_ phonon–plasmon response is modified, which may arise from changes in free-carrier density, plasmon damping, alloy disorder, and local optical-coupling variations near the graded interface.

After incorporating the combined fitting and spatial-sampling uncertainties, the effective transition widths at the p^++^/n^++^ junction are 2α­(TO) = 190 ± 80 nm, 2α­(L_1_) = 170 ± 50 nm, 2α­(TOM) = 120 ± 50 nm, and 2α­(LO_2_) = 130 ± 80 nm. The TOM and LO_2_ modes yield consistent widths within uncertainty, indicating that the InGaP-to-InP compositional transition is reproducibly captured by both alloy-sensitive phonons. In contrast, the broader TO response, together with the comparable L_1_ width within uncertainty, likely reflects additional contributions from lattice disorder and carrier-related phonon–plasmon coupling, respectively, which broaden their Raman intensity profiles across the transition.

We note that these transition centers and widths follow directly from empirical sigmoidal fits to the measured lattice–mode profiles: TOM, TO, and LO_2_, and do not rely on any model-based calibration.

The ordering of the fitted transition centers at the p^++^/n^++^ junction, with L_1_ attenuating first, followed by the TO response and then the TOM/LO_2_ modes, is consistent with the precursor-switching sequence during axial growth. As schematically illustrated in Figure S6, the transition from p^++^-InGaP to n^++^-InP involves maintaining the In precursor flow while abruptly terminating the Ga and Zn precursors and introducing sulfur for n^++^ doping. Under these conditions, residual Ga retained in the Au catalyst and/or along the NW sidewalls can continue to contribute to the local growth front over a finite axial distance. This carry-over can delay the disappearance of the InGaP-related TOM and LO_2_ modes relative to the emergence of the InP TO signal, which appears as soon as InP-rich material begins to form after interruption of the Ga supply. In contrast, the earlier suppression of the L_1_ response relative to the lattice-derived InGaP modes indicates that the phonon–plasmon-coupled Raman signal is modified before the complete loss of the InGaP structural signature. This behavior is consistent with changes in the local carrier/plasmonic environment near the junction, which may arise from carrier depletion, enhanced plasmon damping, alloy disorder, and/or variations in local optical coupling.

The different ordering of the Raman-mode transitions at the two interfaces therefore points to mode-dependent and asymmetric junction evolution governed by the distinct precursor-switching sequences and residual group-III carry-over. This signifies that TOM and LO_2_ are reliable markers of the InGaP-related compositional transition, TO as an indicator of the onset of InP-rich character, and L_1_ as an electronic-sensitive marker of the local carrier/plasmonic environment. Because the L_1_ response can be influenced by carrier density, plasmon damping, alloy disorder, and local optical coupling, it is treated as a semiquantitative indicator of electronic junction evolution rather than as a direct depletion-width probe.

Since the quantitative interpretation of the junction profiles depends on the effective TERS spatial response,^27^ we independently evaluated the near-field response using sharp nanoscale reference systems measured with Ag-coated probes fabricated and operated under conditions comparable to those used for the InP/InGaP NWs. A single-wall carbon nanotube on Au exhibited a G-band response confined to a single 10 nm pixel without far-field subtraction (Figure S7). As an additional benchmark, a CdS/Sb_2_Se_3_ heterostructure measured on the same TERS platform showed a CdS-to-Sb_2_Se_3_ spectral transition within one 10 nm step (Figure S8).^30^ These measurements establish an effective TERS near-field response on the order of 10 nm under comparable operating conditions. However, for the NW line profiles analyzed in Figures 3 and 4, the 30 and 50 nm spatial sampling intervals dominate the uncertainties in the fitted transition centers and widths (Table 2, Supplementary Note 1).

Furthermore, the finite-element simulations in Figure S9 illustrate that, when a tilted Ag-coated TERS tip interacts with a curved NW flank, the near-field enhancement can be displaced toward the tip–sidewall contact region and may produce an apparent lateral broadening of the Raman response at the NW edge. Thus, a NW flank would not necessarily provide a simple, geometry-independent edge-response measurement of the TERS point-spread function. Moreover, the NW-edge simulations in Figure S9 rationalize why a sharp NW contour is not expected in Figure 2. Therefore, in the present analysis, the transition widths are extracted from axial line profiles through the junction regions, away from an intentional transverse edge-response geometry.

While correlative high-resolution transmission electron microscopy coupled with energy-dispersive X-ray spectroscopy (HR-TEM/EDX) would provide a valuable independent benchmark of the elemental composition profile, such data are not available for the same NWs analyzed here, and obtaining them would require destructive preparation of a longitudinal TEM lamella aligned with the axial junctions investigated by TERS. In addition, EDX would provide a thickness-averaged elemental projection, whereas TERS probes the near-surface Raman response and, through the L_1_ mode, also provides sensitivity to the local carrier/plasmonic environment. Therefore, we do not claim an external EDX validation of the compositional profiles in the present work. Instead, the compositional gradients are inferred from the mutually consistent spatial evolution of lattice-derived Raman modes, with TOM used as the primary Raman marker of the InGaP-related compositional boundary and TO/LO_2_ used as supporting composition-sensitive indicators. The mode-selective nature of TERS enables the lattice-derived Raman response to be separated from the carrier/plasmonic L_1_ response. This distinction allows the structural/compositional junction evolution to be assessed from TO, TOM, and LO_2_, while the L_1_ mode provides a complementary electronic-sensitive marker of changes in the local carrier/plasmonic environment, as discussed below and in Figure S5.

The fitted transition parameters for all Raman modes at the p^++^/n^++^ tunnel diode junction are also summarized in Table 2. As with the p/p^++^ interface, COMSOL simulations using the experimentally measured transition widths reveal sloped conduction and valence band edges across the p^++^/n^++^ junction (Figure 4c), deviating from the idealized abrupt case. The resulting graded potential produces a slightly asymmetric and extended barrier profile, motivating the qualitative WKB trend analysis below and illustrating the expected sensitivity of tunnel-barrier probability to the nanoscale interfacial profile.

To place the TERS-derived transition lengths in the context of device-relevant NWs, we compare the L_1_-derived transition widths with independent KPFM measurements performed on shorter InP/InGaP NWs of 2.7 μm length that are representative of the operating tandem devices. KPFM probes the local electrostatic potential rather than composition, yet the potential profiles across both the p/p^++^ and p^++^/n^++^ junctions exhibit smooth sigmoidal variations analogous to those observed in the TERS intensity maps. Accordingly, the KPFM profiles were analyzed using the same sigmoidal model (eq 4), yielding transition widths of 2α = 106 ± 3 nm for the p/p^++^ junction and 2α = 139 ± 5 nm for the p^++^/n^++^ junction (Figure S10). When compared with the L_1_-derived TERS transition widths measured on the longer 8.4 μm NW, 2α = 140 ± 50 nm for the p/p^++^ junction and 2α = 170 ± 50 nm for the p^++^/n^++^ junction, the KPFM central values are smaller, consistent with the shorter, device-relevant NW length. This comparison remains qualitative, because TERS and KPFM probe different physical quantities: phonon–plasmon coupling in TERS versus surface electrostatic potential in KPFM. In addition, KPFM is laterally convoluted by the finite AFM probe response. KPFM is therefore used as a complementary electronic-potential probe supporting the trend of junction grading rather than a direct validation of the L_1_-based carrier-density analysis or of the TERS spatial response.

As a first-order approximation, the experimentally measured TERS transition widths can be rescaled to shorter, device-relevant NWs. For the TOM mode, the extrapolated widths are approximately 28 nm for the p/p^++^ junction and 35 nm for the p^++^/n^++^ junction, whereas for the L_1_ mode the corresponding values are 43 nm (p/p^++^) and 52 nm (p^++^/n^++^). These values provide a conservative estimate of the junction grading in shorter wires. The KPFM measurements, which capture the electronic profile at the surface, qualitatively support this trend: although the widths measured by KPFM are larger than the extrapolated TERS values due to the finite tip size and surface sensitivity, they nonetheless show a systematic decrease with NW length, consistent with the TERS scaling.

Having established the measured spatial evolution of the Raman modes, we next provide a model-assisted estimate of the local free-hole concentration. The L_1_ and LO_1_ mode frequencies are sensitive to free-hole concentration through phonon–plasmon coupling;^46,47^ however, the conversion from Raman frequency to carrier density is model dependent. In particular, the Lindhard–Mermin calibration depends on the plasmon damping parameter Γ.^41^ For the consolidated p^++^ In_0.27_Ga_0.73_P tunnel-diode segment, the (p, Γ) pair was previously determined by Mediavilla et al.^41^ through a full Lindhard–Mermin line-shape fit of the L_1_ band measured on the p^++^-InGaP side of the same tunnel-diode structure — identical nominal composition and doping scheme, NWs from the same growth platform, and the same 532 nm excitation used here — yielding p ≈ 1 × 10^20^ cm^–3^ and Γ = 900 cm^–1^.^41^ Because the full band-shape fit determines the carrier density and the damping simultaneously, it resolves the (p, Γ) degeneracy that affects frequency-only analyses; in the present work no parameter is adjusted, and this published calibration is applied directly to the TERS-measured L_1_ frequencies in the consolidated p^++^ segment. In contrast, within the graded junction regions spatial variations in Γ are plausible, because alloy composition, defect density, and structural disorder may vary across the interface. To evaluate this sensitivity, we calculated the L_1_ frequency–carrier-density calibration for Γ = 700, 900, and 1100 cm^–1^ (Figure S5) and used these curves to generate the corresponding carrier-density profiles (Figure S11). This analysis shows that the absolute carrier concentration, and to some extent the apparent carrier-density gradient, depends on the assumed damping parameter. Therefore, we regard the carrier-density values as model-assisted estimates rather than absolute quantitative measurements. Importantly, the transition centers and transition widths reported in Table 2 are obtained from sigmoidal fits to the integrated Raman intensity profiles and do not depend on the Γ-dependent frequency-to-carrier-density conversion. Nevertheless, the physical interpretation of the L_1_ intensity profile is semiquantitative as this intensity can be affected by carrier density, plasmon damping, alloy disorder, and local optical coupling.

We present in Figure 5 only the results obtained with Γ = 900 cm^–1^, which corresponds to the overdamped regime relevant to this work. We note, however, that the absolute carrier concentration derived from the L_1_ frequency remains model dependent, as variations in Γ directly affect the calibration. Figure 5a traces the Raman shifts of the L_1_ and LO_1_ modes across the p/p^++^ junction, and Figure 5b shows the resulting hole–density profile obtained by calibrating the L_1_ frequency against established doping dependence.^46,47^ Moreover, we also included the nominal hole concentration calculated with COMSOL, shown in blue line in Figure 5b, which follows the same trend that the extracted hole concentration using the peak position of the Raman mode L_1_ and LO_1_. It should be noted that the LO_1_ (InGaP) mode used to estimate the carrier concentration on the InP side does not arise from pure InP, but from the graded junction transition where Ga incorporation begins. In this junction region, the gradual increase in Ga content leads to the emergence of an GaP-like LO (LO_1_) vibration associated with In_*x*_Ga_1–*x*_P, before the L_1_ mode becomes dominant. Consequently, the extracted carrier density represents the mixed-composition region at the onset of InGaP formation rather than in the InP segment. This interpretation is consistent with the nonabrupt nature of the junction and the compositional grading revealed by TERS mapping.

**Figure 5:**
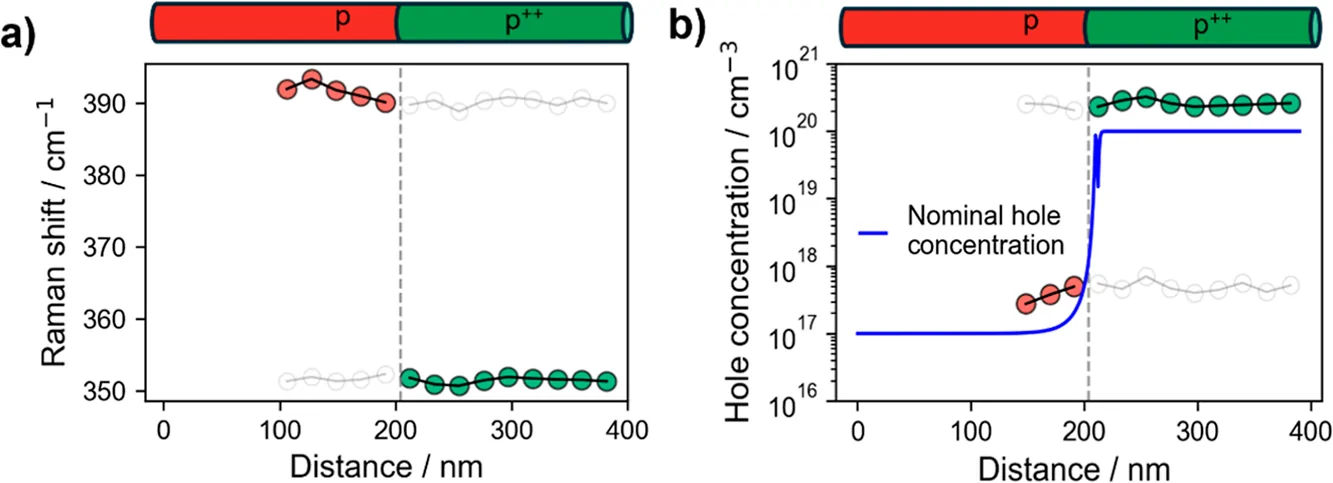
Model-assisted estimation of the local free-hole profile from phonon–plasmon-coupled Raman modes. (**a**) Raman shifts of the LO_1_ and L_1_ modes as a function of position along the NW. The vertical dashed line marks the p/p^++^ junction at *x*_0_ = 204 nm, determined from the sigmoidal fit to the TOM intensity profile. (**b**) Estimated free-hole concentration profile derived from the measured Raman shifts using the representative Lindhard–Mermin calibration with Γ = 900 cm^–1^. For *x* < *x*_0_, the estimate is obtained from the LO_1_ frequency in the graded transition region; for *x* > *x*_0_, it is obtained from the L_1_ frequency in the p^++^ segment. The blue curve shows the nominal hole concentration used in COMSOL for comparison. The absolute carrier concentration is model dependent; the sensitivity to Γ is shown in Figure S11.

Direct electrical characterization of the individual *p*-type segments in these NWs is hampered by nonohmic contacts to the p-side. However, current–voltage measurements on p^++^-InGaP/n^++^-InP NW tunnel diodes grown under the same conditions exhibit clear negative differential resistance,^17,31^ which requires band-to-band tunneling between degenerately doped segments and therefore independently corroborates free-carrier concentrations in the 10^20^ cm^–3^ range in the tunnel-diode segments. We further note that TERS is more surface-sensitive than the far-field micro-Raman measurements used to establish the calibration, so near-surface band bending could in principle shift the apparent carrier density. This does not affect the order-of-magnitude conclusion for the degenerately doped p^++^ segment. Within the graded transition regions, in contrast, the derived carrier-density profile is presented only as a model-assisted consistency check against the nominal doping trend (Figure 5b), not as a quantitative carrier-density map.

Using the representative Γ = 900 cm^–1^ calibration, the L_1_/LO_1_-based analysis gives a model-assisted estimate of the free-hole concentration increasing from approximately 2 × 10^17^ cm^–3^ in the graded *p*-InP/p^++^-InGaP transition region to approximately 1 × 10^20^ cm^–3^ in the p^++^-InGaP segment. The sensitivity analysis in Figure S11 shows that the absolute values depend on the assumed Γ, but the qualitative trend of a strong increase in hole concentration toward the p^++^ segment is retained. The peak value is broadly consistent with the nominal doping level, supporting the physical plausibility of the model-assisted estimate.

In a heavily doped p^++^/n^++^ tunnel junction, the depletion region is expected to be very narrow. The TERS data, however, are not used here to measure that depletion width directly.

The coupled evolution of the local free-hole density and the compositional profile produces an asymmetric response at the two interfaces. This asymmetry is most clearly manifested in the L_1_ mode, owing to its mixed hole-plasmon–phonon character. Using the TOM transition center as the compositional reference and the L_1_ transition center as an electronic-sensitive marker, the relative L_1_–TOM offset magnitudes are Δ*x*_0_ = 80 ± 40 nm for the p/p^++^ transition and Δ*x*_0_ = 150 ± 40 nm for the p^++^/n^++^ transition. The difference between these offset magnitudes, 70 ± 60 nm, suggests an asymmetric electronic response across the two interfaces, consistent with the built-in electric field of the tunnel diode. However, we regard this comparison as semiquantitative because of the combined fitting and spatial-sampling uncertainties. The different L_1_ transition widths at the two interfaces further indicate that the phonon–plasmon Raman response evolves over a broader spatial range than the TOM compositional marker. Because the L_1_ intensity decay may contain inseparable contributions from compositional grading, carrier density, plasmon damping, alloy disorder, and local optical coupling, we interpret these widths as apparent carrier-sensitive Raman attenuation lengths.

The variation in transition width observed for the InP TO mode indicates substantial lattice disorder at the InP/InGaP junctions, arising from both heavy doping and Ga incorporation. Such disorder likely contributes to the reduced quantum efficiency reported for state-of-the-art III–V NW tandem solar cells. To qualitatively illustrate how interfacial grading modifies the tunnel-barrier transparency, we calculated an equilibrium, zero-bias, one-dimensional WKB tunneling factor, *T*, for idealized abrupt and graded band profiles using the measured transition widths as input (full derivation, equations, and the resulting *T*-versus-transition-width trend are provided in the Supporting Information, Supplementary Note 2, and Figure S12).^48,49^ This equilibrium calculation does not include bias-dependent band bending or carrier occupation, interface states or trap-assisted tunneling, recombination losses, or series resistance, and is therefore not a finite-bias tunnel-diode transport simulation. Within this simplified model, increasing the interfacial transition width monotonically and steeply decreases *T*, illustrating that broader compositional grading is expected to widen the effective tunnel barrier and reduce its relative transparency. This qualitative trend underscores the importance of nanoscale characterization of junction profiles, such as that provided by hyperspectral TERS, for guiding tunnel-diode design.

## Conclusions

In summary, herein we used hyperspectral TERS mapping to resolve the nanoscale vibrational and electronic signatures of axial heterojunctions in InP/InGaP NW tandem solar cells. Mode-selective analysis shows that the nominally abrupt p/p^++^ and p^++^/n^++^ junctions are spatially graded rather than atomically sharp. The TOM mode, used as the primary compositional marker, yields structural transition widths of approximately 90–120 nm, while other lattice- and carrier-sensitive modes exhibit broader central widths up to ∼190 nm, reflecting additional sensitivity to alloy disorder, heavy doping, lattice relaxation, and phonon–plasmon coupling. The L_1_ phonon–plasmon mode provides an internal, nanoscale carrier-sensitive Raman probe of the local free-hole environment. Its transition center was found to be offset from the TOM compositional marker by 80 ± 40 nm at the p/p^++^ interface and 150 ± 40 nm at the p^++^/n^++^ interface, indicating an asymmetric carrier-sensitive Raman response across the tunnel diode. However, because the L_1_ response can also be influenced by plasmon damping, alloy disorder, and local optical coupling, these offsets are interpreted as carrier-sensitive signatures of junction evolution rather than direct quantitative measurements of depletion width. The free-hole concentration in the consolidated p^++^ segment, obtained by applying an independently established Lindhard–Mermin calibration^41^ for the same tunnel-diode structure, reaches ∼10^20^ cm^–3^, consistent with the nominal doping and with the negative differential resistance reported for equivalent NW tunnel diodes;^17,31^ within the graded regions, the corresponding carrier-density profile is model-assisted and serves as a consistency check against the nominal doping trend.

As a first-order approximation, rescaling the TERS-derived transition widths to the 2.7 μm NWs examined by KPFM gives nominal junction widths of ∼28–35 nm for the TOM compositional marker and ∼43–52 nm for the L_1_ carrier-sensitive marker. KPFM measurements qualitatively support the same trend of reduced transition length in shorter NWs, although they probe surface electrostatic potential and are subject to tip-induced lateral convolution. Thus, KPFM is used here as a complementary device-scale probe rather than as a validation of TERS spatial response. Furthermore, a simplified equilibrium one-dimensional WKB calculation, detailed in the Supporting Information, indicates that the tunnel-barrier transparency is highly sensitive to the interfacial transition width, with broader grading expected to reduce it. This calculation is a qualitative, equilibrium comparison of interface-profile sensitivity and is not used to predict device current.

Overall, these results demonstrate that real axial tunnel junctions in III–V NW tandem photovoltaics are governed by gradual and asymmetric chemical transitions, with a carrier-sensitive Raman component that evolves differently from the structural composition profile. Such grading must therefore be explicitly considered in tunnel-diode design and in the interpretation of carrier transport in multijunction NW devices. More broadly, this work establishes mode-selective hyperspectral TERS as a chemically specific nanoscale method for mapping structural and compositional grading in III–V NW junctions, while L_1_ phonon–plasmon response provides complementary carrier-sensitive Raman contrast: quantitative in the consolidated p^++^ segment, where an independent calibration^41^ and device-level electrical evidence^17,31^ support the extracted hole density, and model-assisted within the graded regions, where its conversion to electronic parameters remains model-dependent.

## Methods

### Nanowire Growth

InP/InGaP NWs were synthesized following the procedure described by Hrachowina *et al*.^9^ In brief, a hexagonal array of Au catalyst particles (500 nm pitch) evaporated through an e-beam evaporator from Temescal Systems, USA was patterned on 2-in. n-type InP(111)B wafers (InPACT, France) by Displacement Talbot Lithography (Eulitha, Switzerland), metal evaporation, and lift-off. The wafers were then diced into 9 × 11 mm substrates. Growth was carried out in a metal–organic vapor-phase epitaxy (MOVPE) reactor (Aixtron 200/4, Germany) with total H_2_ flow 13 L min^–1^, 100 mbar. A low-temperature prenucleation step at 280 °C using trimethylindium^50^ (TMIn) and phosphine (PH_3_) was followed by annealing at 550 °C to remove surface oxides. Axial NW growth was initiated at 440 °C with TMIn and PH_3_; hydrogen chloride (HCl) was introduced to suppress radial deposition.^51^ Dopant incorporation was controlled using H_2_S for n-type segments and diethylzinc (DEZn) for p-type segments to form the targeted p/p^++^ and p^++^/n^++^ segments.^9^ Triethylgallium (TEGa) was added to achieve controlled Ga concentration both in the p^++^ region of the tunnel diode and in the top n-i-p InGaP subcell.

### TERS Setup and Measurements

TERS experiments were performed on a LabRAM Soleil Raman spectrometer integrated with an AFM (Horiba Scientific, France). Excitation was provided by a 532 nm solid-state laser. Spectral resolution of the Raman spectrometer was 2.4 cm^–1^. Ag-coated TERS probes were fabricated in-house from commercial Si AFM tips (AppNano, USA). The probes were thermally oxidized, ozone-cleaned, and coated with 120 nm Ag in a vacuum evaporator at <10^–6^ mbar with a slow evaporation rate of 0.05 nm s^–1^. The effective near-field spatial response of this probe configuration was independently assessed using single-wall carbon nanotubes (SWCNTs) deposited on an Au substrate and measured under comparable experimental conditions (Figure S7). The SWCNT G-band response was confined to a single 10 nm pixel, indicating a TERS spatial response on the order of 10 nm, limited by the sampling interval. An additional benchmark from a CdS/Sb_2_Se_3_ heterostructure acquired on the same TERS platform also showed an abrupt spectral transition within one 10 nm pixel (Figure S8).^30^

Hyperspectral TERS mapping of the p/p^++^ and p^++^/n^++^ junctions was performed with spatial step sizes of Δ*x* = 30 nm and Δ*x* = 50 nm, respectively. These sampling intervals were chosen as a practical compromise between spatial response, map size, and spectral quality. Because the InP/InGaP NWs were mechanically transferred onto the Au-coated substrate rather than rigidly fixed to it, appreciable drift occurred during extended hyperspectral TERS acquisition. The use of 30–50 nm steps therefore enabled mapping over a sufficiently large field of view, at least 0.25 × 0.25 μm^2^, while maintaining an acceptable signal-to-noise ratio within 10 s spectral integration time.

Possible laser-induced heating during TERS mapping was evaluated from the spectral stability of the hyperspectral data sets. The TERS maps were acquired with excitation laser power of 600 μW at the sample, corresponding to an incident intensity of approximately 8.9 × 10^2^ μW μm^–2^. During mapping, the typical spectral signatures for significant laser-induced heating including systematic Raman peak shifts, line width broadening, increased spectral background, signal degradation, or the appearance of additional peaks were not observed, thus indicating that laser-induced heating or photoinduced modification does not measurably affect the TERS spectra under the present acquisition conditions.

Confocal Raman spectra under top illumination were acquired in a similar length NW perpendicularly oriented with respect to the electric field. A laser power of 2.5 mW was used with 40 s acquisition time, and a pinhole of 200 μm.

### Data Processing

TERS spectra were processed in LabSpec software (Horiba Scientific, France). A linear baseline was first removed to eliminate background contributions, after which a light polynomial smoothing filter was applied to improve signal-to-noise ratio without distorting peak shapes. Peaks were then fit with a multipeak Lorentzian model, including the InP TO mode (290–315 cm^–1^), the InGaP TOM mode (320–349 cm^–1^), the L_1_ phonon–plasmon mode (350–359 cm^–1^), the LO_2_ mode (360–379 cm^–1^), and the LO_1_ mode (385–395 cm^–1^). Integrated peak areas from the fits were used to generate 2D intensity maps for each vibrational mode. Figure 2f and i were generated with ImageJ. To assess axial variations, a line profile was extracted along the actual NW orientation. The 2D TERS maps in Figure 2 are shown in the original acquisition coordinates (hence some NWs appear diagonally aligned). The effective spatial sampling along the NW axis was determined from the Euclidean distance between consecutive pixels along the extracted line.

Pixels from the grid lines immediately adjacent to the extracted profile were also included, projected onto the NW axis, to increase the sampling density along the profile. For the uncertainty analysis (Supplementary Note 1), the acquisition step sizes (Δ*x* = 30 nm for the p/p^++^ map and 50 nm for the p^++^/n^++^ map) were conservatively used as the spatial-sampling intervals, without credit for the increased profile point density.

No spectrum-to-spectrum intensity normalization was applied to the TERS data before peak fitting, and the fitted peak areas were not normalized after fitting. The fitted integrated peak areas therefore retain the relative intensity variations measured within each hyperspectral map. Furthermore, all spectra within a given map were acquired under identical experimental conditions, including constant laser power, integration time, optical alignment, and feedback conditions. No large, random pixel-to-pixel intensity excursions indicative of loss of tip enhancement, unstable acquisition or spurious signals from atmospheric carbon contaminants were observed. However, the absolute TERS intensity did exhibit modest spatial fluctuations, as expected for near-field Raman measurements, where the detected signal can be influenced by small variations in local tip–sample coupling, NW curvature, and optical coupling. Therefore, the fitted peak areas are not interpreted as absolute Raman cross sections or as quantitatively comparable intensities between different maps or different vibrational modes. Instead, the integrated intensities are used as relative, mode-specific observables within each individual map.

Peak parameters were refined sequentially rather than simultaneously: for each mode, the peak position was first fitted with the width and amplitude held fixed, the amplitude was then fitted with the position fixed, and the width was refined last, iterating this sequence until stable values were obtained. Transition centers *x*_0_ and effective widths 2α were obtained from least-squares fits of eq 4 to the integrated-intensity axial profiles, using the effective spatial sampling intervals along the NW axis determined as described above. Fitting and spatial-sampling uncertainties were combined in quadrature as described in Supplementary Note 1. Additional data are available from the corresponding authors upon reasonable request.

### KPFM Measurements

Amplitude-modulated Kelvin probe force microscopy measurements were performed using a Bruker Dimension Icon atomic force microscope (Bruker Corporation, USA) under ambient conditions. Conductive SCM-PIT-V2 probes (Bruker) with PtIr-coated Si cantilevers were used in tapping mode. Each line was recorded in two passes: the first scan acquired the topography in regular tapping mode, while in the second one the tip followed the height profile with a lift height of ∼20 nm under applied AC and DC bias to map the local surface potential. Data were processed using Gwyddion software by applying a second-order polynomial background subtraction with spatial masking and a line-by-line correction to minimize scanning artifacts.

## Supplementary Material



## Notes

Comprehensive characterization and analysis of InP/InGaP tandem NWs. AFM topography and height profiles provide precise nanowire length, height, and lateral width measurements. Hyperspectral TERS mapping of In_*x*_Ga_1–*x*_P L_1_ and TOM modes across p/p^++^ and p^++^/n^++^ junctions revealing compositional and electronic transitions at the nanoscale, with stacked TERS spectra along the nanowire axis, highlighting mode-dependent sensitivity to chemical composition and carrier concentration. Calculations of L_1_-mode frequency versus hole density using the Lindhard–Mermin model illustrating the model-dependent sensitivity of the phonon–plasmon Raman response to the local free-carrier concentration. Vapor–liquid–solid growth precursor profiles detailing the formation of each nanowire segment, explaining the experimentally observed junction grading. Kelvin probe force microscopy maps and extracted potential profiles on shorter, device-relevant NWs with complementary qualitative surface-potential trends that support the scaling behavior observed in TERS. Illustrative equilibrium WKB analysis of the p^++^/n^++^ tunnel-diode junction (Supplementary Note 2), showing the qualitative dependence of the calculated barrier transparency on the interfacial transition width (PDF)
