# Supplementary material for: Mode-Selective Tip-Enhanced Raman Spectroscopy Reveals Asymmetric Chemical and Carrier-Sensitive Raman Grading in Photovoltaic III–V Tandem Nanowire Junctions
Source: ACS Nano. 2026 Oct 12;20(42):28419–31. doi: 10.1021/acsnano.6c10214 (PMC13718602; doi:10.1021/acsnano.6c10214)
Supplement: Supplementary file 1 [file nn6c10214_si_001.pdf]

Supporting Information

Mode-Selective Tip-Enhanced Raman Spectroscopy Reveals Asymmetric Chemical and Carrier-Sensitive Raman Grading in Photovoltaic III–V Tandem Nanowire Junctions

Vanessa Hinojosa-Chasiquiza,[1] *Jose Luis Pura,*[1] *Siiri Bienz,*[2] *Yue Zhao,*[3,4] *Jordi Antoja-Lleonart,*[5] *Magnus Borgström,*[3,6] *Francesc Pérez-Murano,*[5] *Sergio Muñoz de Baena*[1]*,* *Renato Zenobi,*[2] *Naresh Kumar,*[2]* *and Jorge Serrano*[1,5,7]*

[1]Gds-Optronlab, Dpto. Física de la Materia Condensada, Universidad de Valladolid, Paseo de Belén 19, 47011, Valladolid, Spain

[2]Department of Chemistry and Applied Biosciences, ETH Zürich, 8093 Zürich, Switzerland

[3]Nano Lund and Division of Solid-State Physics, Lund University, Box 118, 221 00 Lund, Sweden

[4]Hexagem AB, Ole Römers väg 1H, 223 63 Lund, Sweden

[5]Institute of Microelectronics of Barcelona (IMB−CNM, CSIC), Campus UAB, 08193 Bellaterra, Barcelona, Spain

[6]Wallenberg Initiative Materials Science for Sustainability, Department of Solid-State Physics, Lund University, 221 00 Lund, Sweden

[7]Meeting Life LLC, 1210 Washington Ave. Ste. 213, 33139 Miami Beach, Florida, USA

*E-mail: naresh.kumar@org.chem.ethz.ch, jorge.serrano@imb-cnm.csic.es

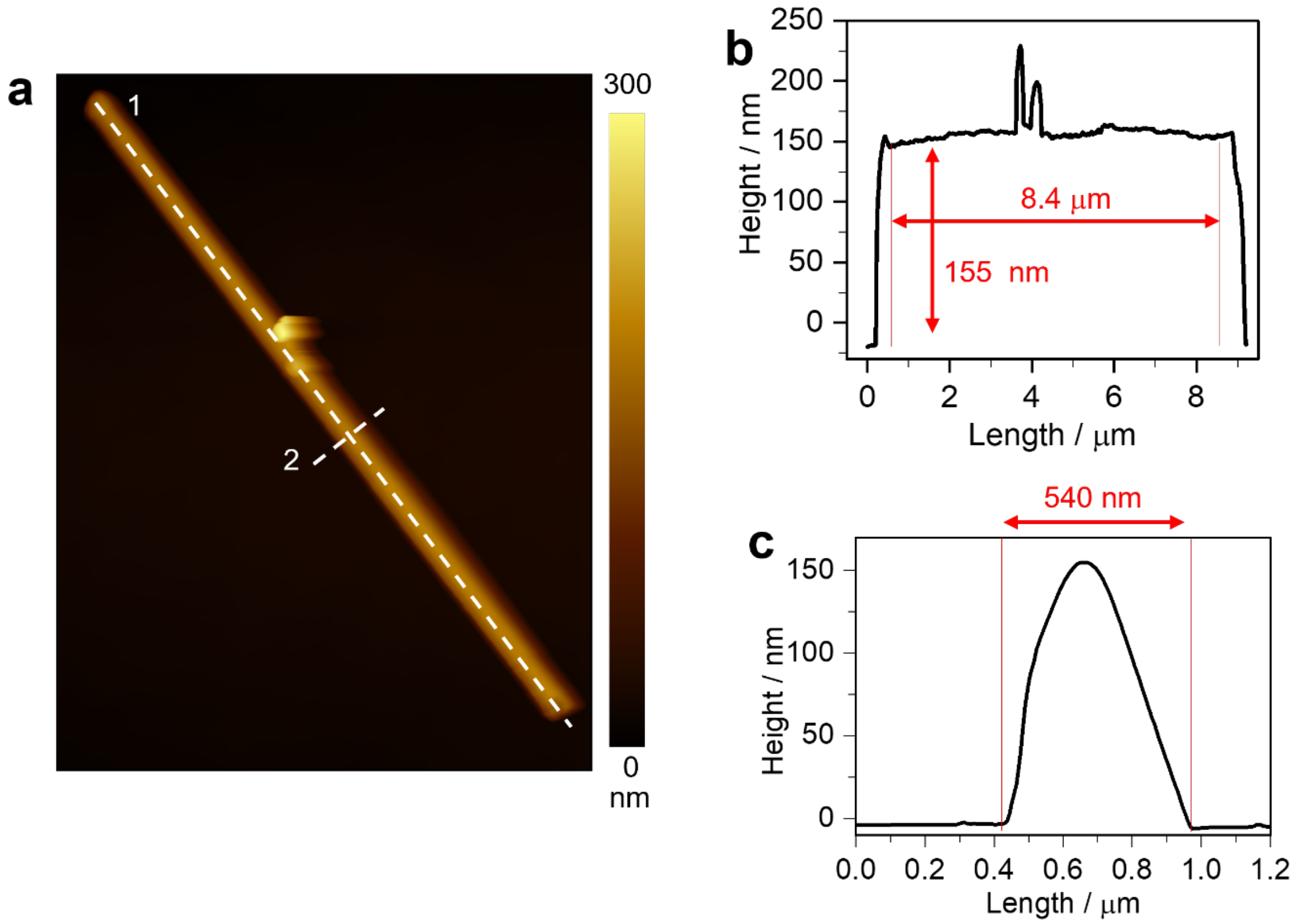

Figure S1. Atomic-force microscopy (AFM) topographic characterization of the InP/InGaP nanowire investigated in this work. **(a)** AFM topography image of the selected nanowire. **(b)** Axial height profile, profile 1, extracted along the nanowire axis indicated in panel (a). This profile gives a nanowire length of ~8.4 μm and a height of ~155 nm. **(c)** Transverse height profile, profile 2, extracted across the nanowire axis indicated in panel (a). The apparent lateral width is ~540 nm. This lateral width reflects the convolution of the AFM tip size and the nanowire (NW) width. This also reflects that the AFM tip is structurally inclined by design with respect to the vertical axis, thus leading to a broader and asymmetric profile. Therefore, the vertical height provides a more direct estimate of the nanowire height.

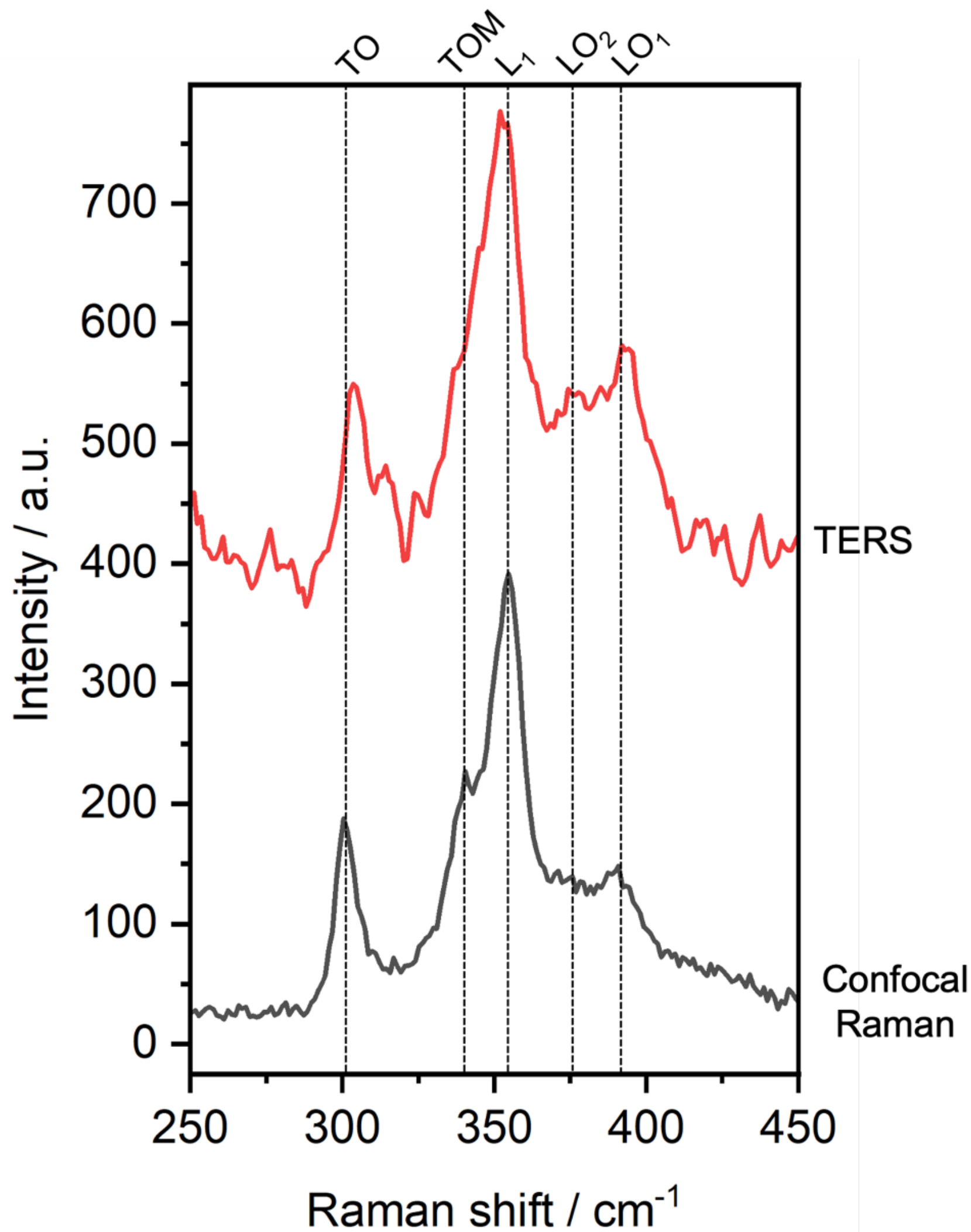

Figure S2. Representative tip-enhanced Raman spectroscopy (TERS) spectrum of the measured NW, red Conventional confocal micro-Raman spectrum, black, acquired from a representative InP/InGaP NW under top-illumination confocal conditions using 532 nm excitation, 2.5 mW laser power at the sample, and 40 s integration time. The spectrum shows the same characteristic Raman modes observed in the TERS measurements, including the TO mode of InP, and the TOM, L_1, LO_2 and LO_1 modes of InGaP. Note that although a slight shift in the peak positions is observed in the TERS measurements, the values are still within the 2.4 cm^{-1} spectral resolution of our spectrometer. The relative peak intensities in the TERS and conventional micro-Raman spectra are not directly comparable because the two techniques probe different optical volumes and enhancement conditions. Nevertheless, the agreement in peak positions confirms that the intrinsic vibrational response of the InP/InGaP NW is preserved in TERS measurements.

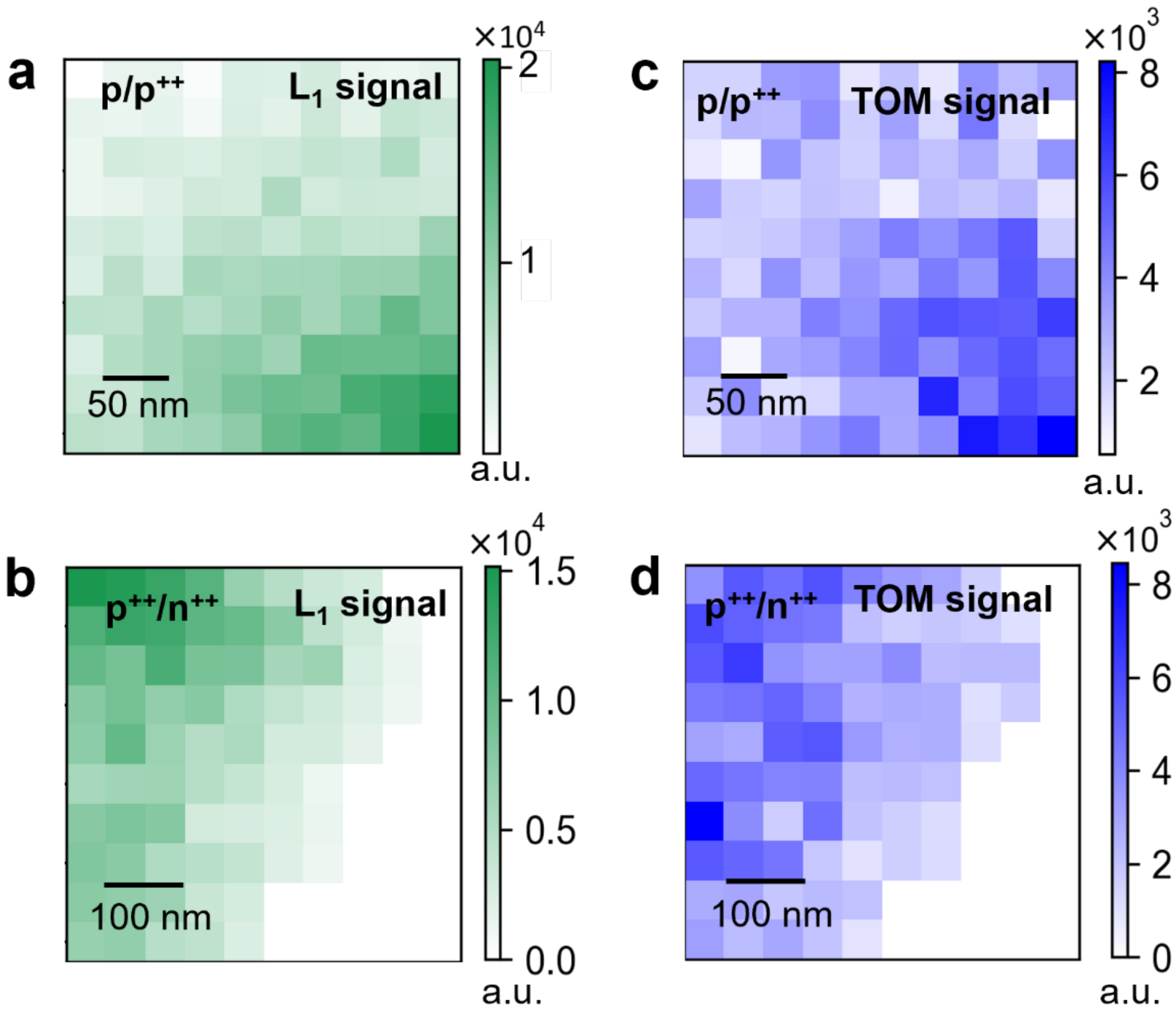

Figure S3. TERS intensity maps of the $In_xGa_{1-x}P$ L_1 and TOM modes across the (a, c) p/p^{++} and (b, d) p^{++}/n^{++} junctions. The TOM mode provides a lattice-derived Raman marker of the InGaP-related compositional transition, whereas the L_1 mode reflects the local carrier/plasmonic response. Their correlated spatial evolution shows that the carrier/plasmonic response changes across the same junction region as the composition-sensitive TOM signal, although L_1 is not treated as a direct elemental-composition marker.

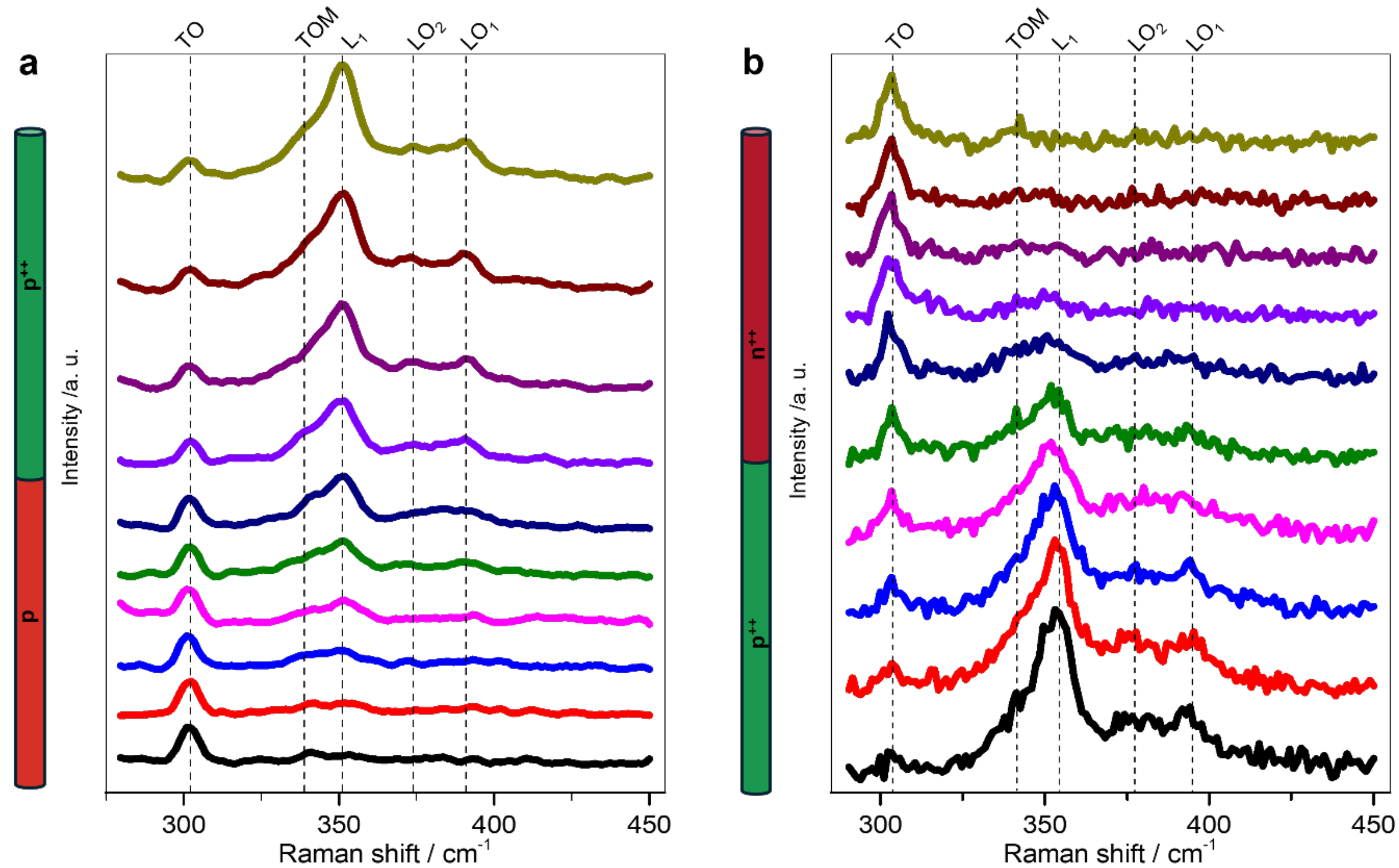

Figure S4. Stacked TERS spectra acquired along the nanowire (NW) growth axis across the two heterojunctions. (a) p/p^{++} junction and (b) p^{++}/n^{++} tunnel diode junction. Each stacked spectrum corresponds to a single spatial point along the axial line scan obtained through the junction region. The left-side schematics depict the respective segment configurations within the NW. Characteristic Raman modes are marked by dashed lines: the InP transverse optical (TO) mode, and the $In_xGa_{1-x}P$ longitudinal optical phonon–plasmon coupled mode (L_1) and transverse optical mixed (TOM) mode. The gradual spectral evolution and peak intensity exchange between TO and L_1 modes reveal the compositional and electronic grading across each junction. In the p/p^{++} junction (a), the TO intensity decreases while the L_1 mode emerges toward the p^{++} side, indicating progressive Ga incorporation and Zn doping. Conversely, in the p^{++}/n^{++} junction (b), the TO mode strengthens and the L_1 mode diminishes across the transition, consistent with the onset of S doping and a local suppression or modification of the phonon–plasmon coupling near the junction. The stacked representation emphasizes the nanoscale continuity of the Raman features and the non-abrupt nature of both junctions.

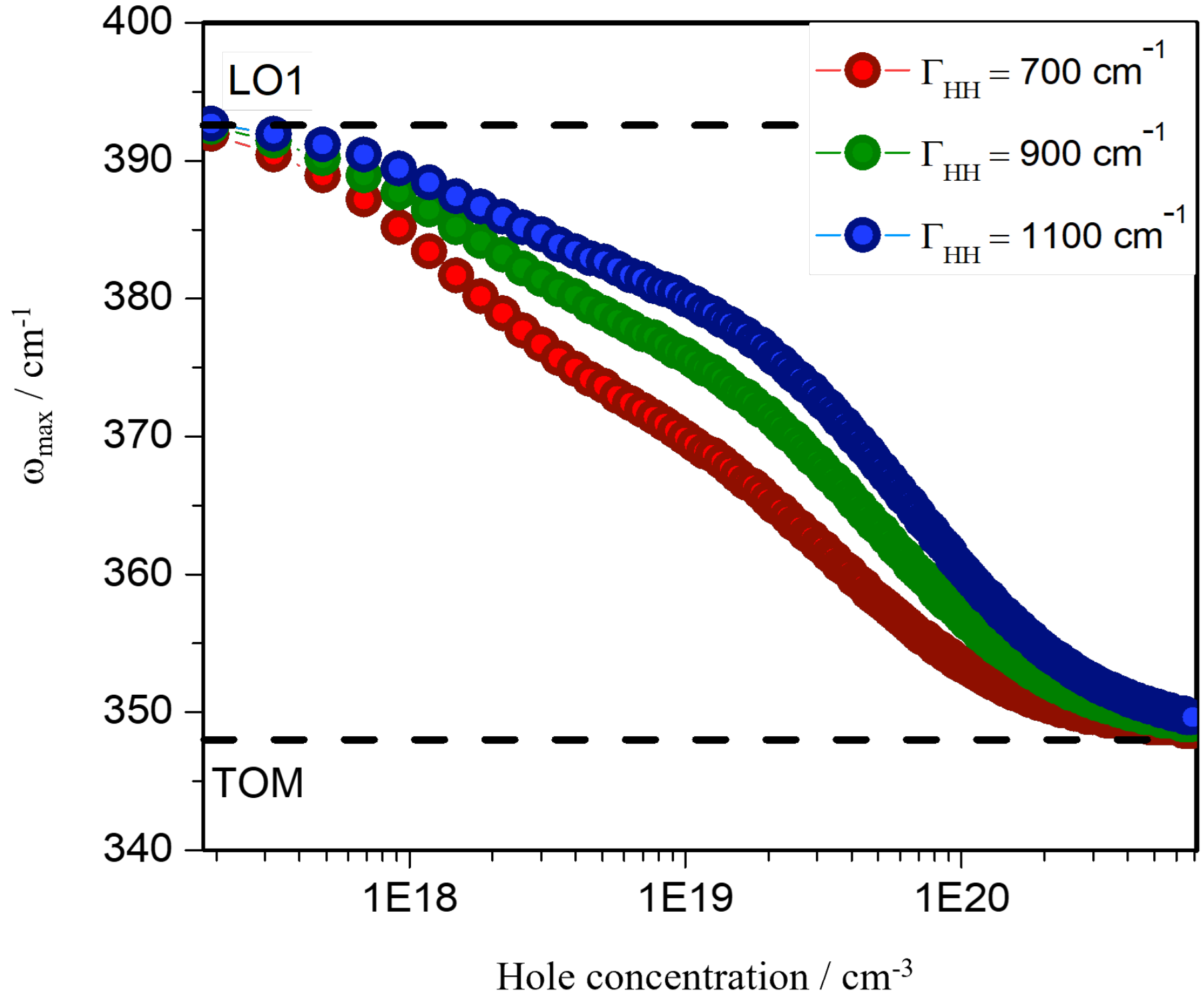

Figure S5. Sensitivity of the L_1 mode frequency-to-hole-concentration calibration to the plasmon damping parameter. Calculated dependence of the L_1 mode frequency on free-hole concentration using the Lindhard–Mermin model for $\Gamma = 700$, 900, and 1100 cm^{-1}, adapted from Mediavilla *et al.*[1] The $\Gamma = 900$ cm^{-1} curve corresponds to the representative overdamped regime used for the carrier-density estimate in Figure 5. The spread among the curves illustrates the model dependence of the frequency-to-carrier-density conversion and the sensitivity of the inferred carrier density to the assumed plasmon damping parameter.

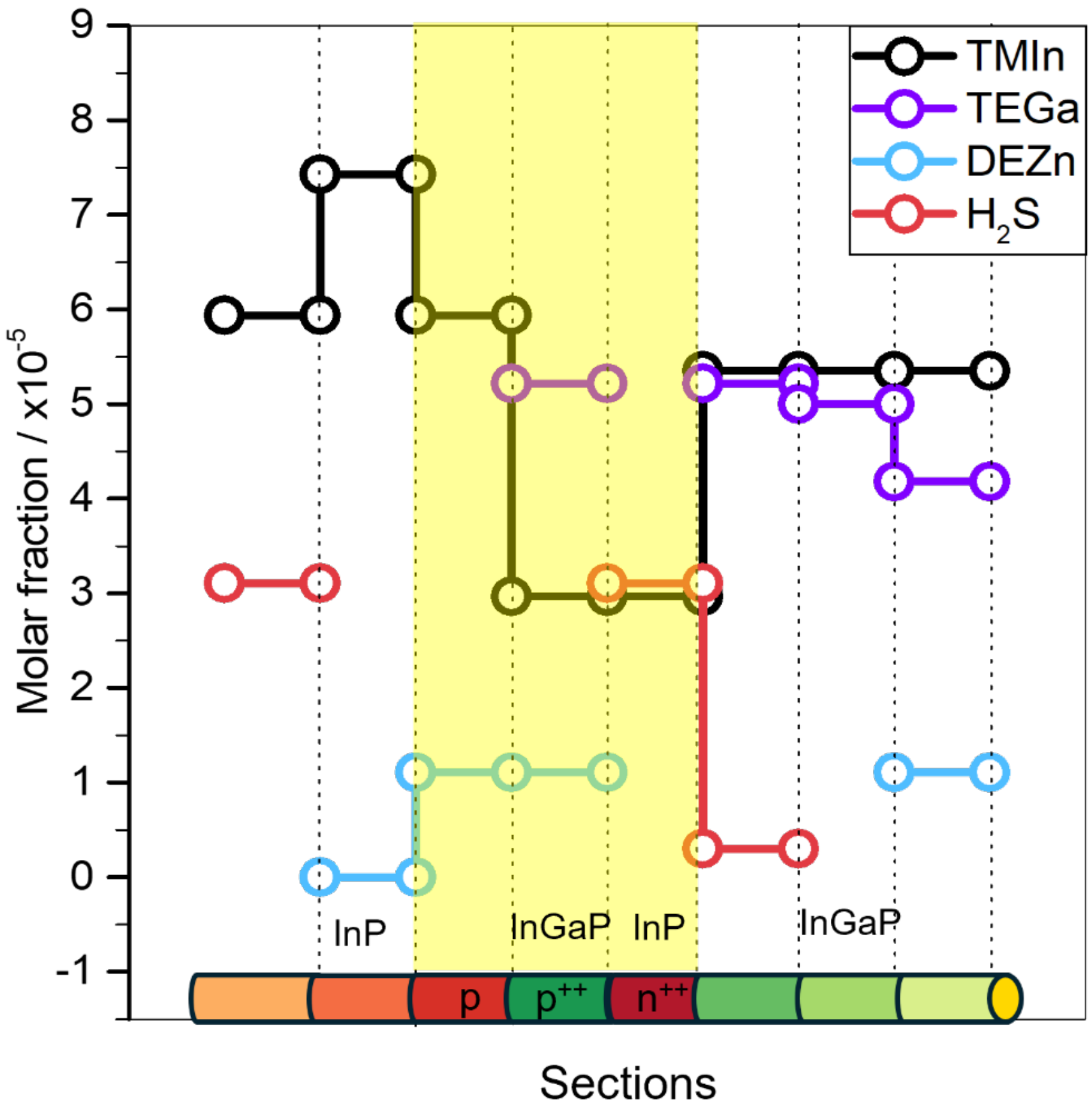

Figure S6. Schematic representation of precursor molar fraction profiles used during the vapor–liquid–solid (VLS) growth of the tandem InP/InGaP nanowire. The yellow area highlights the region of interest for this work. The x-axis indicates the successive nanowire segments (not to scale), aligned with the structural sketch shown above, while the y-axis represents the relative molar fractions of the gaseous precursors employed: trimethylindium (TMIn), triethylgallium (TEGa), diethylzinc (DEZn), and hydrogen sulfide (H_2S). The plotted traces illustrate the sequence of precursor flow modulation that defines the formation of the p-InP, p^{++}-$In_{0.27}Ga_{0.73}P$, and n^{++}-InP segments, corresponding respectively to the p/p^{++} and p^{++}/n^{++} junctions analyzed in the main text. The precursor switching sequence provides the growth context for the mode-dependent compositional and doping-related gradients inferred from TERS. The final junction profiles are expected to be influenced not only by the nominal precursor flows, but also by catalyst carry-over, sidewall diffusion, and dopant incorporation kinetics.

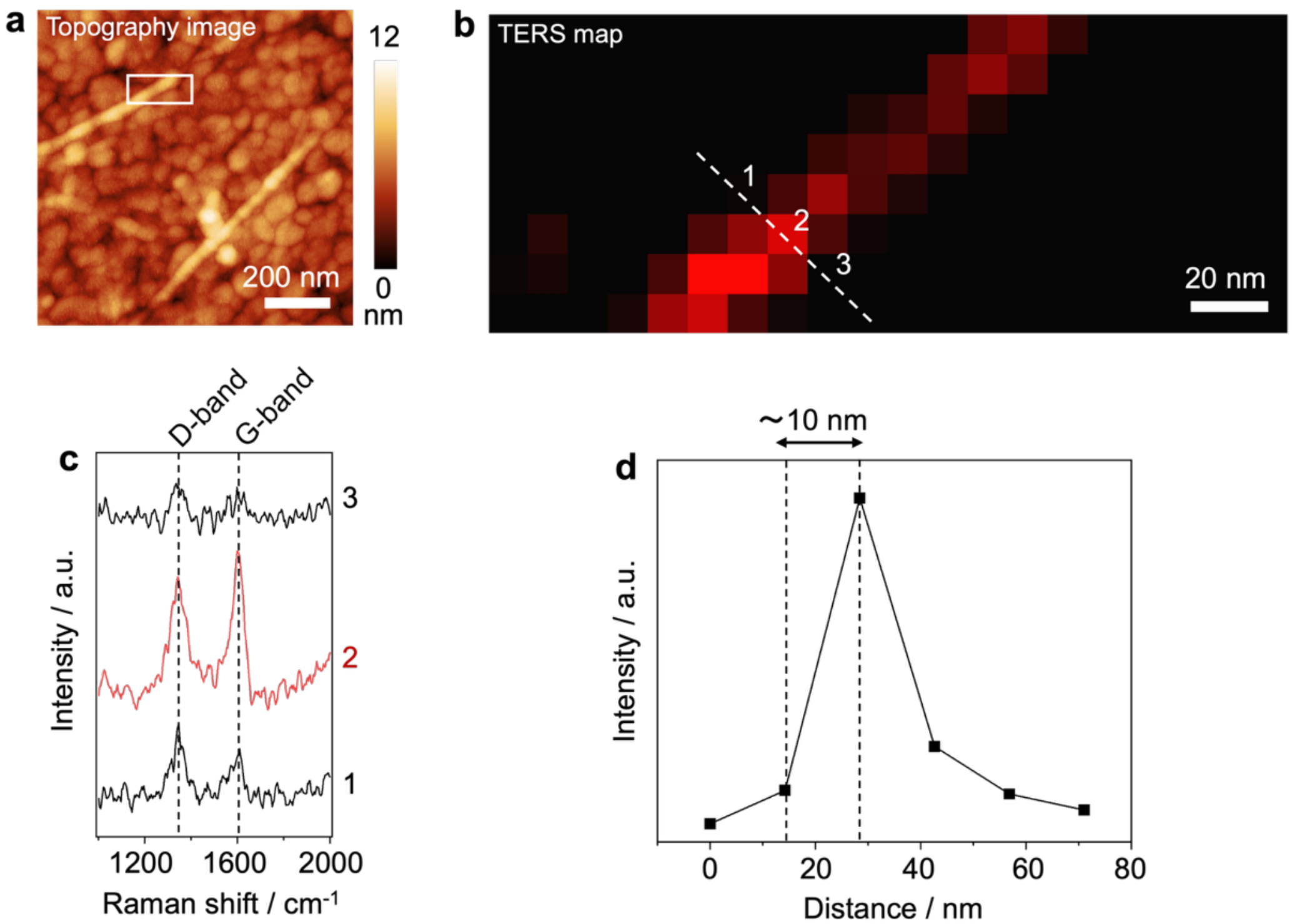

Figure S7. Assessment of the TERS spatial response using single-wall carbon nanotubes (SWCNTs) on a Au substrate. **(a)** AFM topography image of SWCNTs deposited on Au. **(b)** TERS map of the SWCNT region marked in panel (a), reconstructed from the integrated G-band intensity at ~1600 cm^{-1}. The map was acquired with a 10 nm step size and 1 s integration time per spectrum. The maximum G-band response is confined to a single pixel, and no far-field subtraction was applied. **(c)** Representative TERS spectra acquired at positions 1–3 in panel (b). The strong G-band signal at position 2 is assigned to the near-field-enhanced SWCNT response, whereas the weaker signals at positions 1 and 3 are attributed mainly to residual far-field/background contributions. **(d)** Line profile extracted along the dashed line in panel (b). The abrupt intensity rise within one scan step indicates an effective TERS spatial response of less than 10 nm, limited by the sampling step size of the reference measurement.

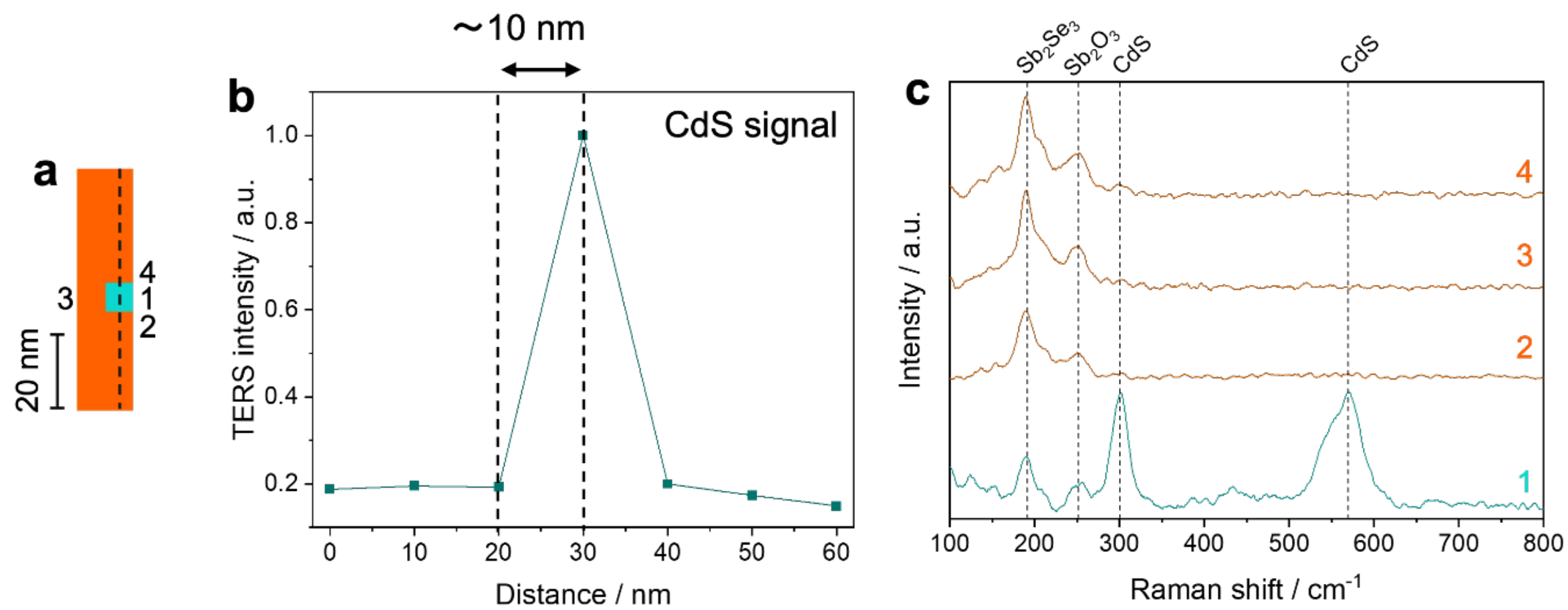

Figure S8. Verification of TERS spatial resolution using a CdS/Sb_2Se_3 heterostructure. **(a)** Overlay of hyperspectral TERS maps reconstructed from the characteristic CdS and Sb_2Se_3 Raman signals, shown in green and orange, respectively. The spatial distribution reveals CdS interdiffusion into the Sb_2Se_3 layer. The TERS map was acquired with a 10 nm step size and an integration time of 1 s per spectrum. **(b)** Corresponding normalized TERS intensity profile of the CdS signal (560 cm^{-1}) extracted along the line indicated in panel (a). **(c)** Representative TERS spectra acquired at the positions marked in panel (a). The abrupt change in CdS TERS intensity within one scan step, together with the spectral transition from CdS-dominated to Sb_2Se_3-dominated signatures within a single pixel, demonstrates a TERS spatial resolution on the order of 10 nm, limited by the mapping step size. Panels (a–c) are adapted with permission from Bienz *et al*.[2]

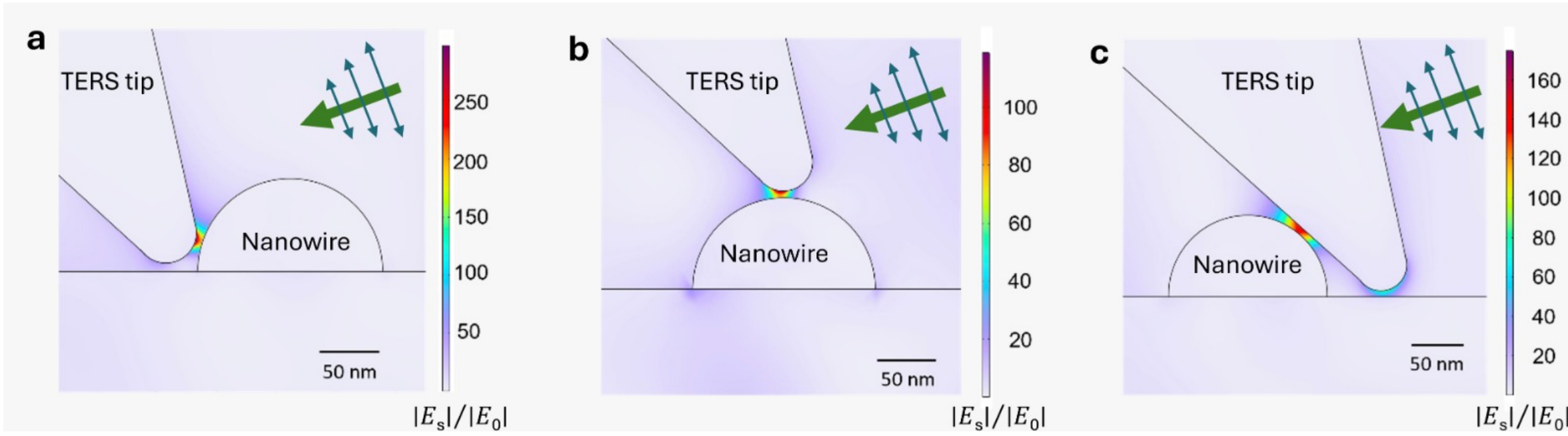

Figure S9. Finite-element (FE) simulations of the TERS tip–NW near-field interaction**.** (a–c) FE simulations of the normalized scattered electric-field amplitude, $|E_s|/|E_0|$, for a tilted TERS tip positioned at different lateral locations across the NW cross section. FE simulations were performed using COMSOL Multiphysics. The TERS tip is located at the left flank, top region, and right flank of the NW in panels (a), (b), and (c), respectively. The tilted tip geometry was used to reproduce the experimental bent-tip configuration, in which the tip axis is oriented perpendicular to the incident optical field under side illumination as depicted in panels (a-c). The simulated field distributions reveal strong nanoscale confinement of the tip-enhanced near field at the tip–nanowire junction. Furthermore, the interaction between the curved nanowire surface and the TERS tip shaft can locally amplify the optical field at the NW flanks, leading to an apparent broadening of the TERS spatial response at the NW edges. These simulations qualitatively illustrate that a curved NW flank may not provide a simple, geometry-independent edge-response measurement of the TERS point-spread function.

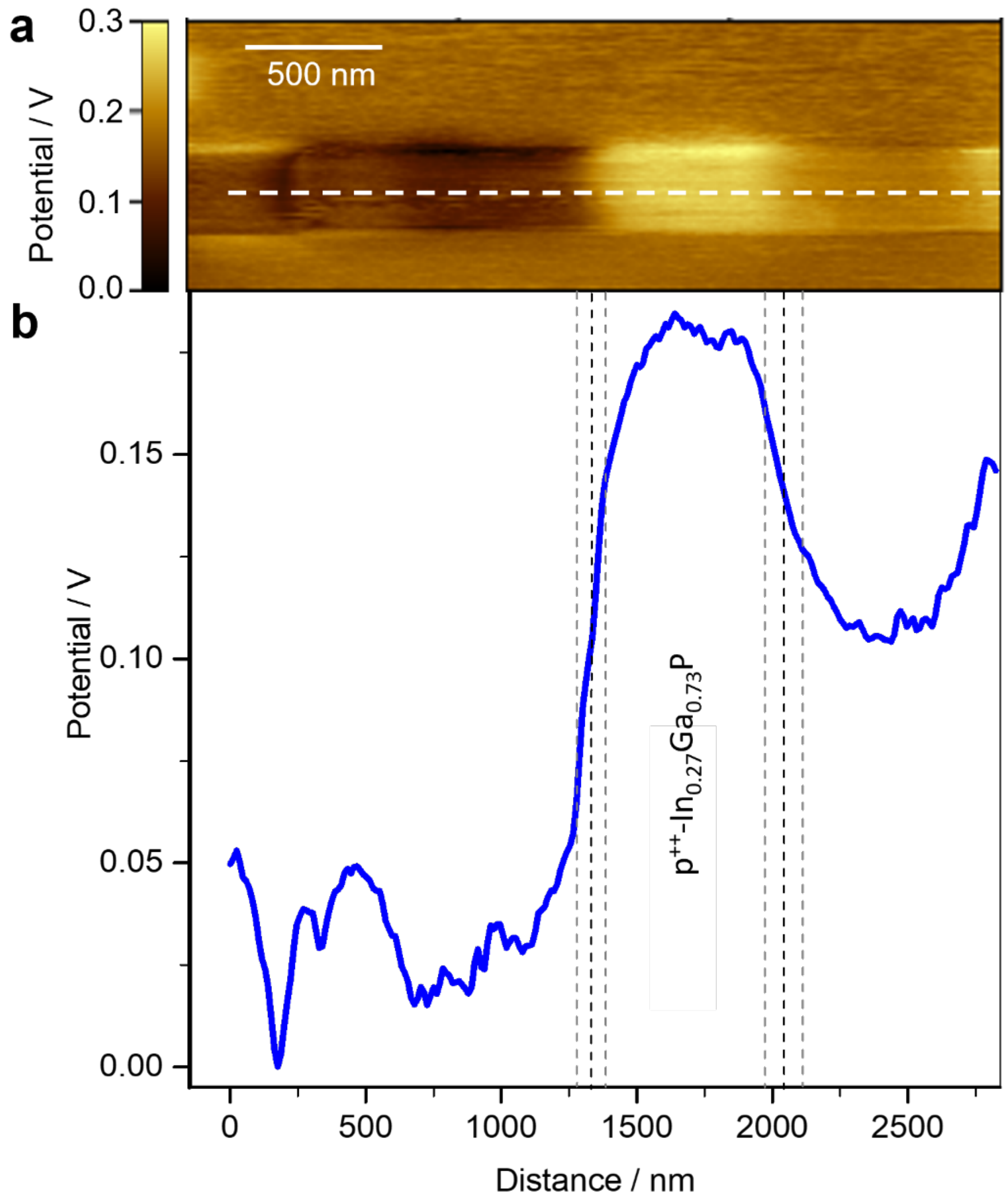

Figure S10. (a) Kelvin probe force microscopy (KPFM) surface potential map of a 2.7 µm-long InP/$In_xGa_{1-x}P$ (x = 0.91 for top cell and x = 0.27 for the p^{++} segment) nanowire tandem device. The white dashed line indicates the line scan along which the potential profile was extracted. **(b)** Corresponding potential profile showing the different regions along the nanowire. The dashed area marks the p^{++}-InGaP section of ~700 nm. Gray dashed lines indicate the 2α transition widths obtained from sigmoid fits. The maximum surface potential in (b) reaches ~0.15 V, lower than the ~0.3 V scale in (a), because the highest-potential region lies outside the line scan.

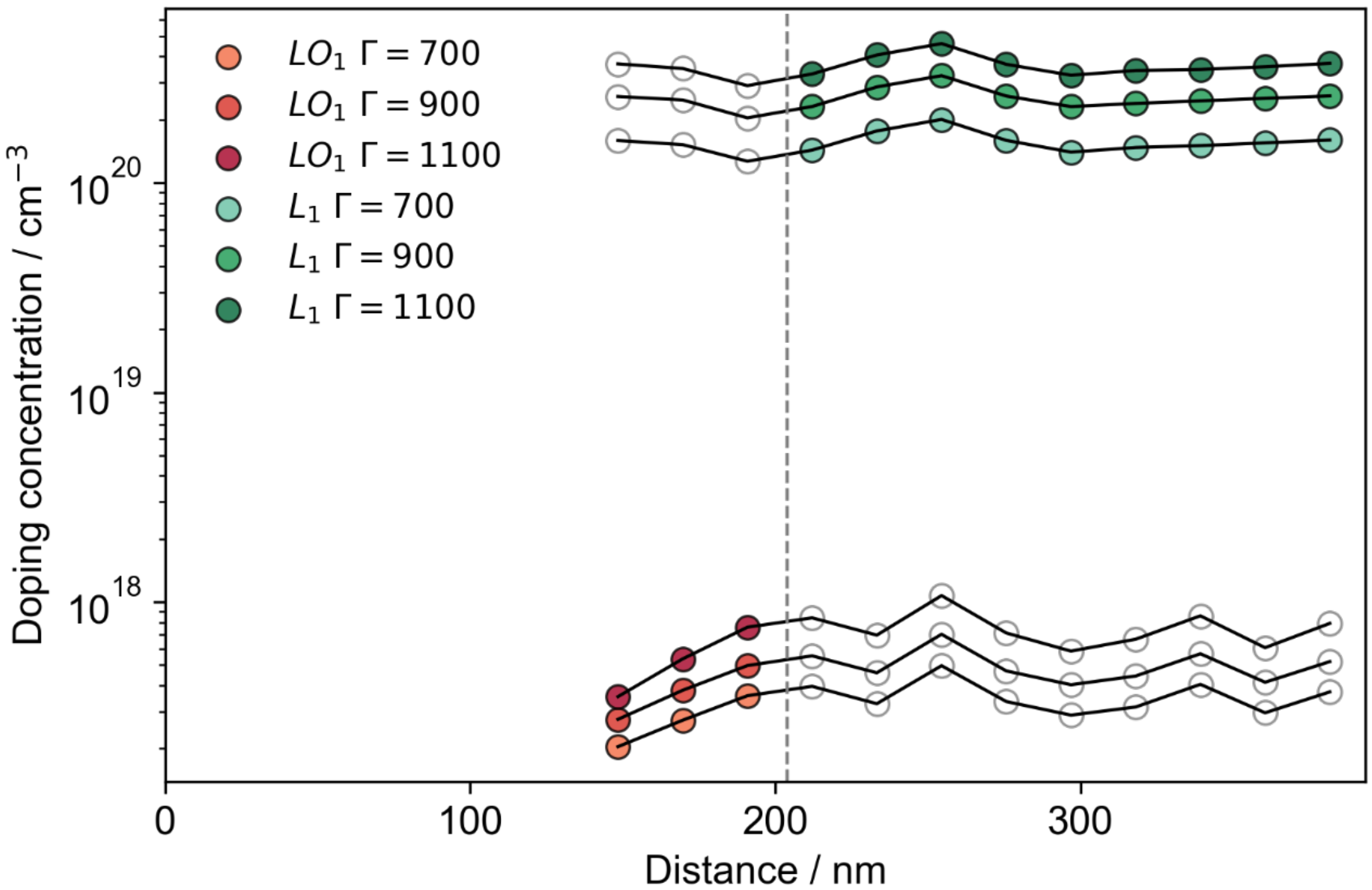

Figure S11. Sensitivity of the estimated free-hole concentration profile to the assumed plasmon damping parameter. Free-hole concentration profiles along the NW derived from the measured LO_1 and L_1 Raman shifts using Lindhard–Mermin calibration curves calculated for Γ = 700, 900, and 1100 cm^{-1}. The LO_1 frequency is used for the graded p-side transition region, while the L_1 frequency is used for the p^{++} region. The spread among the profiles illustrates the systematic dependence of the extracted carrier concentration on Γ. These profiles are therefore used as a sensitivity check for the model-assisted carrier-density estimate. The transition centers and widths reported in Table 2 are obtained from integrated-intensity fits and are not determined by this Γ-dependent conversion.

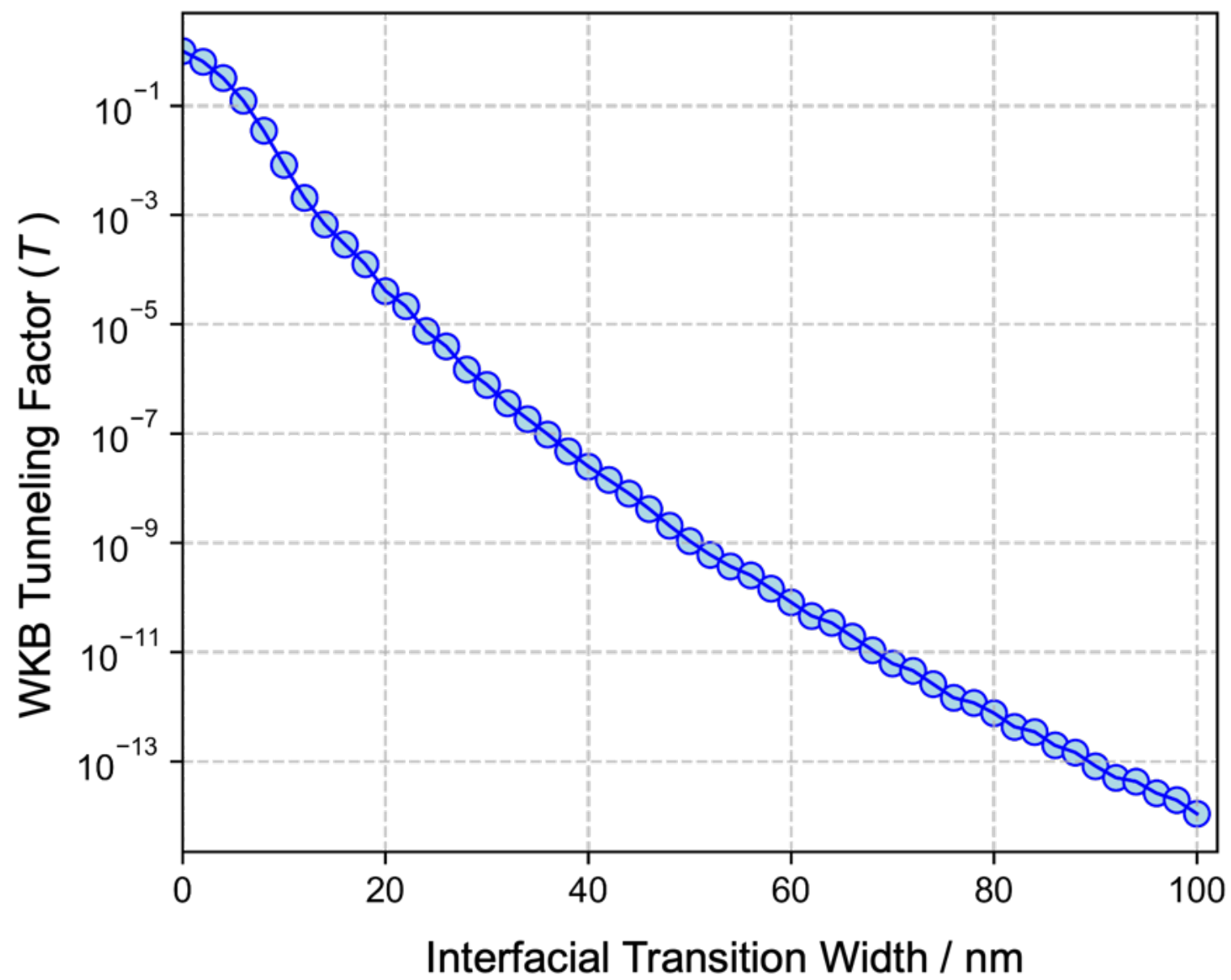

Figure S12. Calculated equilibrium one-dimensional WKB tunneling factor, *T*, as a function of interfacial transition width for the p^{++}/n^{++} tunnel diode junction. The values were obtained from simulated axial zero-bias band-edge profiles and are used to compare idealized abrupt and compositionally graded junction profiles. Note that this WKB treatment is a simplified equilibrium model rather than a finite-bias tunnel-diode transport simulation: it does not include bias-dependent band bending, carrier injection or carrier occupation effects, interface states or trap-assisted tunneling, recombination, or series resistance. Consequently, we do not interpret *T* as a calculated tunnel-diode current, current density, quantum efficiency, or device-performance metric. The absolute values of *T* depend on the assumed effective mass, band-edge profile, transition width, and energy reference. Therefore, this plot is used only to illustrate the qualitative trend that increasing junction grading increases the WKB action and reduces the relative tunnel-barrier transparency.

Table S1. Evidence classification of quantities discussed in this work. This table clarifies which quantities are used to support the main conclusions versus which are presented as secondary, model-assisted, or purely illustrative.

Quantity	Status	Used for main conclusions?	Caveat
Raman spectra, peak positions, integrated intensities	Directly measured	Yes	TERS enhancement/local coupling affects absolute intensities; not normalized across maps
TOM/TO/LO_2 transition centers and widths	Empirical fit to lattice-mode intensity profiles	Yes	Raman-inferred compositional grading, not EDX-calibrated composition
L_1 transition center/width	Empirical fit to phonon–plasmon Raman response	Secondary	Carrier-sensitive but also affected by damping, disorder, and optical coupling
L_1/LO_1 carrier-density profile	Model-assisted Lindhard–Mermin estimate	No, consistency check only	Depends on assumed Γ and local material parameters
KPFM transition widths	Complementary surface-potential measurement	Qualitative support only	Different NW length, surface sensitivity, tip convolution
WKB tunneling factor, T	Illustrative equilibrium calculation	No	Not a finite-bias current or device model

Rows classified as directly measured or empirically fitted ("Yes" in the third column) support the manuscript's main conclusions independently of the rows classified as model-assisted or illustrative ("No"); removing the latter rows entirely would not alter the classification or support of the former.

Supplementary Note 1

Uncertainty analysis for TERS-derived transition centers and transition widths

The transition centers x_0 and effective transition widths 2α reported in Table 2 were extracted from TERS line profiles measured across the p/p^{++} and p^{++}/n^{++} junctions. The integrated Raman intensity profiles of the relevant modes were fitted using a sigmoidal function:

$$I(x) = I_{\min} + \frac{I_{\max} - I_{\min}}{1 + \exp\left[\pm \frac{x - x_0}{\alpha}\right]}, \qquad \textbf{(S1)}$$

where $I_{\min}$ and $I_{\max}$ are the limiting intensities, x_0 is the transition center, and α describes the abruptness of the transition. The effective transition width is defined as

$$w = 2\alpha. \qquad \textbf{(S2)}$$

The $\pm$ sign in equation S1 accounts for either increasing or decreasing Raman intensity across the junction, depending on the vibrational mode and interface direction.

The formal fitting uncertainties obtained from the nonlinear least-squares fits are denoted as $u_{\text{fit}}\ (x_0)$ and $u_{\text{fit}}\ (2\alpha)$. These uncertainties quantify the statistical confidence of the fitted parameters for the measured intensity profile. However, because the TERS line profiles are acquired with finite spatial sampling, the fitting uncertainty alone does not fully describe the experimental uncertainty in the extracted interface position or transition width. Specifically, the p/p^{++} and p^{++}/n^{++} junction maps were acquired with spatial step sizes of $\Delta x = 30$ nm and $\Delta x = 50$ nm, respectively. These sampling intervals were chosen as a practical compromise between spatial resolution, map size, and spectral quality. Because the InP/InGaP NWs were mechanically transferred onto the Au substrate rather than rigidly fixed to it, appreciable drift occurred during extended hyperspectral TERS acquisition. The use of 30–50 nm steps therefore enabled mapping over a sufficiently large field of view, at least $0.25 \times 0.25\ \mu m^2$, while maintaining an acceptable signal-to-noise ratio within 10 s spectral integration time.

Although the TERS near-field has a characteristic size of approximately 10 nm under comparable experimental conditions, the line profiles analyzed here were sampled more coarsely than this near-field size. Therefore, the spatial sampling interval represents the dominant contribution to the uncertainty in the extracted transition parameters when the formal fitting uncertainty is smaller than, or comparable to, the step size.

For the transition center x_0, the true interface center may lie between two adjacent sampled points. We therefore assign a conservative spatial-sampling uncertainty equal to one-half of the step size:

$$u_{\text{samp}}(x_0) = \frac{\Delta x}{2}. \qquad \textbf{(S3)}$$

Thus,

$$u_{\mathrm{samp}}(x_0) = 15\ \mathrm{nm}$$

for the p/p^{++} junction and

$$u_{\mathrm{samp}}(x_0) = 25\ \mathrm{nm}$$

for the p^{++}/n^{++} junction.

The total uncertainty in x_0 is then calculated by combining the fitting uncertainty and the sampling uncertainty in quadrature:

$$u_{\mathrm{tot}}(x_0) = \sqrt{u_{\mathrm{fit}}(x_0)^2 + u_{\mathrm{samp}}(x_0)^2} = \sqrt{u_{\mathrm{fit}}(x_0)^2 + \left(\frac{\Delta x}{2}\right)^2}. \qquad \textbf{(S4)}$$

For the effective transition width 2α, a more conservative spatial-sampling uncertainty is used. Because 2α corresponds to a spatial width rather than a single position, it is affected by the localization uncertainty on both sides of the transition. We therefore assign a sampling contribution equal to one full spatial step:

$$u_{\mathrm{samp}}(2\alpha) = \Delta x. \qquad \textbf{(S5)}$$

The total uncertainty in 2α is therefore

$$u_{\mathrm{tot}}(2\alpha) = \sqrt{u_{\mathrm{fit}}(2\alpha)^2 + u_{\mathrm{samp}}(2\alpha)^2} = \sqrt{u_{\mathrm{fit}}(2\alpha)^2 + (\Delta x)^2}. \qquad \textbf{(S6)}$$

This treatment intentionally provides a conservative uncertainty estimate. A purely statistical treatment of the finite pixel spacing as a uniform distribution would lead to smaller standard uncertainties, but here we use the half-step and full-step criteria to avoid overestimating the spatial precision of the TERS-derived interface parameters.

The calculation procedure is illustrated below for representative cases. For the TOM mode at the p/p^{++} junction, the fit gives

$$x_0 = 204\ \mathrm{nm},\, u_{\mathrm{fit}}(x_0) = 10\ \mathrm{nm}.$$

Since $\Delta x = 30$ nm,

$$u_{\mathrm{tot}}(x_0) = \sqrt{(10\ \mathrm{nm})^2 + (15\ \mathrm{nm})^2} = 18\ \mathrm{nm}. \qquad \textbf{(S7)}$$

For the corresponding transition width,

$$2\alpha = 92\text{ nm}, u_{\text{fit}}(2\alpha) = 30\text{ nm}.$$

The total uncertainty is

$$u_{\text{tot}}(2\alpha) = \sqrt{(30\text{ nm})^2 + (30\text{ nm})^2} = 42\text{ nm} \approx 40\text{ nm}. \qquad \textbf{(S8)}$$

For the TOM mode at the p^{++}/n^{++} junction, the fit gives

$$x_0 = 355\text{ nm}, u_{\text{fit}}(x_0) = 10\text{ nm}.$$

Since $\Delta x = 50$ nm,

$$u_{\text{tot}}(x_0) = \sqrt{(10\text{ nm})^2 + (25\text{ nm})^2} = 27\text{ nm} \approx 30\text{ nm}. \qquad \textbf{(S9)}$$

For the corresponding transition width,

$$2\alpha = 116\text{ nm}, u_{\text{fit}}(2\alpha) = 20\text{ nm},$$

and therefore

$$u_{\text{tot}}(2\alpha) = \sqrt{(20\text{ nm})^2 + (50\text{ nm})^2} = 54\text{ nm} \approx 50\text{ nm}. \qquad \textbf{(S10)}$$

The same procedure was applied to all modes listed in Table 2.

The near-field size of the TERS probe was not included as an independent uncertainty term in equation S4 and S6, because the near-field response is already embedded in the measured intensity profiles. This response defines the local excitation and collection volume, and therefore contributes to the overall spatial response of the experiment. However, the NW line profiles analyzed here were acquired with spatial steps of 30 and 50 nm, which are substantially larger than the independently validated ~10 nm near-field response. Thus, for the extracted transition centers x_0 and effective transition widths 2α, the finite spatial sampling represents the dominant additional source of uncertainty beyond the formal fitting error.

To evaluate whether the ~10 nm near-field response could significantly broaden the measured transition widths, we performed a simple point-spread function (PSF) sensitivity analysis. Approximating both the instrumental response and the transition profile as Gaussian contributions gives

$$w_{\text{obs}}^2 \approx w_{\text{int}}^2 + w_{\text{PSF}}^2, \qquad \textbf{(S11)}$$

where w_{obs} is the measured transition width, w_{int} is the estimated intrinsic width, and w_{PSF} is the effective TERS spatial response. For the narrowest measured transition, $w_{\text{obs}} = 90$ nm, using $w_{\text{PSF}} = 10$ nm gives $w_{\text{int}} = 89.4$ nm, corresponding to a change of less than 1 nm. Even under a highly conservative assumption in which the effective spatial response is taken to be comparable to the map sampling interval, the resulting change in the fitted widths remains smaller than the combined uncertainties reported in Table 2. Therefore, the uncertainty in x_0 and 2α is dominated by spatial sampling rather than by the independently validated TERS near-field size.

The measured transition widths, which are substantially larger than the ~10 nm near-field response as well as the spatial sampling of TERS maps, therefore support the conclusion that the observed profiles reflect real compositional and electronic grading rather than instrumental broadening alone.

Supplementary Note 2

Equilibrium one-dimensional WKB tunneling-factor calculation (illustrative, qualitative model)

To qualitatively illustrate how interfacial grading modifies the tunnel-barrier transparency, we calculated an equilibrium one-dimensional WKB tunneling factor, T, for idealized abrupt and graded band profiles,[3]

$$T \approx \exp\big(-S(E)\big) = \exp\left(-2\int_{x_1}^{x_2} \kappa(x,E)dx\right) \quad , \tag{S12}$$

where x_1 and x_2 are the classical turning points defining the barrier region, $\kappa(x,E)$ is the evanescent wavevector, and the approximation is valid for actions $S(E) \gg 1$, (satisfied here). The WKB tunneling factors reported here are computed under equilibrium (zero-bias) band alignments. Because the WKB calculation uses one-dimensional equilibrium, zero-bias band-edge profiles, it is not a finite-bias tunnel-diode transport simulation. It does not include bias-dependent band bending or carrier occupation, interface states or trap-assisted tunneling, recombination losses, or series resistance. Therefore, we use the resulting WKB tunneling factors only as qualitative, model-dependent indicators of how interfacial grading changes the tunnel-barrier transparency. Using the two-band reduced mass m_r:[4]

$$\frac{1}{m_r} = \frac{1}{m_c} + \frac{1}{m_v} \quad , \tag{S13}$$

where m_c and m_v are the effective masses of the conduction and valence bands, respectively, the wavevector can be written as

$$\kappa(x,E) = \sqrt{\frac{2m_r}{\hbar^2}\left(U(x)-E\right)} \approx \sqrt{\frac{2m_r}{\hbar^2}\big(E_c(x)-E+E-E_v(x)\big)} = \sqrt{\frac{2m_r}{\hbar^2}\big(E_c(x)-E_v(x)\big)}\,, \tag{S14}$$

The primary objective of the present calculation is to illustrate the qualitative trend in the WKB tunneling factor as the interfacial transition width is varied, rather than to extract absolute tunneling currents or predict device performance. The WKB framework provides a deliberately simplified way to isolate the structural effect of grading on the equilibrium tunnel barrier, while recognizing that quantitative prediction of device operation would require full finite-bias device-level modeling. The calculated T as a function of the interfacial transition width for the p^{++}/n^{++} tunnel diode junction is presented in Figure S12. Within this simplified equilibrium model, T decreases steeply and monotonically as the interfacial transition width increases. This trend reflects the increase in the WKB action as grading produces a thicker and more extended barrier. We therefore interpret Figure S12 as a qualitative demonstration that a broader junction grading is expected to make direct tunneling less favorable. This result underscores the importance of nanoscale characterization of junction profiles, such as that provided by hyperspectral TERS, for guiding tunnel-diode design.

Within the same simplified equilibrium WKB framework, the measured graded p^{++}/n^{++} profile gives a nominal model tunneling factor of $T_{\mathrm{Gradual}} = 1.53 \times 10^{-23}$, whereas the ideal abrupt reference profile gives $T_{\mathrm{Abrupt}} = 1.02 \times 10^{-8}$. These absolute values are model-dependent and cannot be compared directly with finite-bias tunnel currents or used to infer quantum efficiency. Rather, their contrast illustrates the relative sensitivity of the WKB barrier transparency to the assumed interface

profile. Applying the same model to shorter, device-relevant transition widths shifts the calculation toward higher *T*, qualitatively consistent with the expectation that sharper junctions are more favorable for tunneling. These results highlight the necessity of reliable nanoscale characterization of graded junctions as well as the potential of hyperspectral TERS mapping to guide junction engineering and performance optimization in NW tandem solar cells.

Supplementary references

(1) Mediavilla, I.; Pura, J. L.; Hinojosa, V. G.; Galiana, B.; Hrachowina, L.; Borgström, M. T.; Jimenez, J. Composition, Optical Resonances, and Doping of InP/InGaP Nanowires for Tandem Solar Cells: A Micro-Raman Analysis. *ACS Nano* **2024**, *18*, 10113–10123.

(2) Bienz, S.; Spaggiari, G.; Calestani, D.; Trevisi, G.; Bersani, D.; Zenobi, R.; Kumar, N. Nanoscale Chemical Analysis of Thin Film Solar Cell Interfaces Using Tip-Enhanced Raman Spectroscopy. *ACS Appl. Mater. Interfaces* **2024**, *16*, 14704–14711.

(3) Razavy, M. *Quantum Theory of Tunneling*, 2nd ed.; World Scientific: Singapore, 2013.

(4) Kane, E. O. Zener Tunneling in Semiconductors. *J. Phys. Chem. Solids* **1960**, *12*, 181–188.
